# Multimodal imaging-guided NIR-II photo-gas nanoplatform amplifies PD-1 blockade for synergistic TNBC therapy

**DOI:** 10.7150/thno.133453

**Published:** 2026-06-17

**Authors:** Linlin Song, Meixu Chen, Huiling Wang, Xin Wang, Tianyue Xu, Heqing Zhang, Shuwen Ran, Liwen Huang, Yingying Ma, Zhihui Liu, Zihan Xu, Yujie Zhao, Zichang Liu, Yong Luo, Xiujun Yu, Yulan Peng, Hubing Shi, Jing Jing, Xiujing He

**Affiliations:** 1 Department of Medical Ultrasound, West China Hospital, Sichuan University, Chengdu, Sichuan, P. R. China, 610041.; 2 Institute of Breast Health Medicine, State Key Laboratory of Biotherapy, West China Hospital, Sichuan University and Collaborative Innovation Center, Chengdu, Sichuan, P. R. China, 610041.; 3 Breast Center, West China Hospital, Sichuan University, Chengdu, Sichuan, P. R. China, 610041.; 4 Department of Oncology, Yingshan County People’s Hospital, Nanchong, Sichuan, P. R. China, 637000.

**Keywords:** gas therapy, photothermal therapy, quantum dots, immune checkpoint blockade, theranostic nanoplatform

## Abstract

**Rationale:**

Triple-negative breast cancer (TNBC) remains one of the most aggressive subtypes due to its poor immunogenicity and resistance to systemic therapies.

**Methods:**

Here, we report a multifunctional NIR-II light-triggered theranostic nanoplatform, termed LQPO, that integrates liposome, Ti_3_C_2_ quantum dots (QDs), perfluorohexane (PFH), and ozone to enable multimodal imaging-guided photo-gas synergistic therapy. The QDs, embedded within the liposomal bilayer, act as highly efficient NIR-II photothermal transducers, while the PFH core serves as a liquid-gas phase-change medium capable of dissolving and releasing ozone under localized heating.

**Results:**

Upon NIR-II irradiation, LQPO produced strong photothermal and photoacoustic signals for real-time photoacoustic (PA) and contrast-enhanced ultrasound (CEUS) imaging, accompanied by vaporization of the PFH core and burst release of ozone. This spatiotemporally coordinated cascade induced potent oxidative stress and hyperthermia, synergistically driving GSDME-dependent pyroptosis and immunogenic cell death (ICD). The resulting “in situ vaccination” effect remodeled the tumor immune microenvironment and primed tumors for PD-1 blockade therapy. *In vivo*, NIR-II-activated LQPO achieved efficient tumor accumulation, strong PA/CEUS imaging contrast, and pronounced inhibition of both primary and abscopal tumors when combined with αPD-1 therapy. No significant systemic toxicity was observed, confirming its favorable biosafety.

**Conclusions:**

Overall, this study establishes a single-laser-activated nanoplatform that unifies real-time multimodal imaging and photo-gas synergistic therapy, and converts localized treatment into a systemic antitumor immune response upon integration with checkpoint inhibition.

## Introduction

Triple-negative breast cancer (TNBC) represents a highly difficult-to-treat subtype of breast cancer, primarily owing to its pronounced aggressiveness, limited availability of actionable therapeutic targets, and frequent chemoresistance and recurrence 1, 2. Although the integration of immune checkpoint inhibitors (ICIs) with neoadjuvant chemotherapy has recently improved outcomes for a subset of patients, the overall response rate remains suboptimal 3, 4. One major obstacle is the immunologically “cold” tumor microenvironment (TME) of TNBC, where T-cell infiltration is limited and antigen presentation is often impaired 5. Accordingly, strategies aimed at reshaping TNBC from an immunologically “cold” tumor into a “hot” tumor have attracted increasing attention as a promising approach to improve therapeutic responsiveness.

Among emerging local modalities that can remodel the TME, photothermal therapy (PTT) and oxidative stress-based therapies that generate reactive oxygen species (ROS) are particularly attractive 6, 7. PTT kills tumor cells by converting light energy into heat, with the advantage of localized and controllable treatment. ROS-mediated strategies, such as gas therapy, work through a different mechanism: they increase oxidative stress in metabolically abnormal cancer cells and thereby cause lipid peroxidation, protein damage, and DNA injury 8, 9. Mechanistically, PTT and ROS therapy are highly complementary: PTT is effective even in hypoxic regions and can improve local perfusion and oxygenation, while ROS-based killing is more efficient in well-oxygenated, metabolically stressed microdomains 10. Both approaches can also contribute to immunogenic cell death (ICD). This process is usually accompanied by calreticulin exposure, adenosine triphosphate (ATP) release, and high mobility group box 1 (HMGB1) release, although PTT and ROS-mediated therapy likely initiate these signals through different forms of stress, namely heat shock and oxidative stress 8, 11. Rather than directly guaranteeing effective antitumor immunity, ICD primarily functions as an *in situ* vaccination signal that promotes tumor antigen release and facilitates dendritic cell recruitment and antigen presentation. In the context of TNBC, however, this priming process is frequently restrained by a strongly immunosuppressive microenvironment enriched with regulatory T cells, myeloid-derived suppressor cells, and dysfunctional antigen-presenting cells, thereby limiting spontaneous T-cell activation and effector function. Accordingly, local PTT-ROS treatment establishes a permissive immune context by enhancing tumor antigen availability and cytotoxic T-cell infiltration, thereby enabling effective immune checkpoint blockade 12, 13.

To fully exploit such synergistic local therapies, real-time feedback on drug distribution and treatment activation is crucial. Conventional nanomedicines usually lack robust intra-treatment imaging, making it difficult to precisely define the irradiation field, optimize treatment time, or confirm that therapeutic activation actually occurs within the tumor. Theranostic nanoplatforms that integrate deep-tissue imaging with on-demand therapy under a single clinically relevant external trigger are therefore highly desirable. In particular, combining photoacoustic imaging (PAI) and contrast-enhanced ultrasound (CEUS) imaging within one system can provide complementary information: PAI offers high-resolution mapping of photothermal agents and vascular anatomy, while CEUS enables real-time visualization of phase-change processes and gas generation through the formation of echogenic microbubbles, thereby facilitating accurate treatment guidance, activation monitoring, and early therapeutic response assessment 14-16.

**Figure 1:**
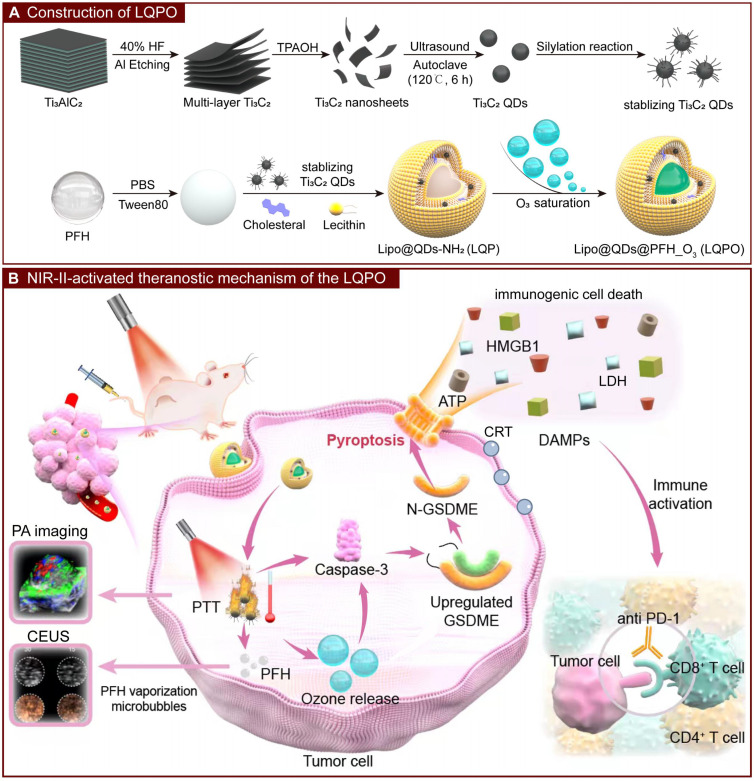
** Construction and NIR-II-activated theranostic mechanism of the LQPO nanoplatform.** (A) Preparation of LQPO. (B) NIR-II-triggered cascade. Upon tumor accumulation, NIR-II irradiation activates Ti_3_C_2_ quantum dots (QDs) to produce photoacoustic (PA) signals and photothermal heating. Upon NIR-II irradiation, QDs efficiently convert light to heat, triggering perfluorohexane (PFH) vaporization and on-demand ozone release, while simultaneously enabling real-time photoacoustic and contrast-enhanced ultrasound imaging. Combined photothermal and oxidative stress promotes tumor pyroptosis and immunogenic cell death, enhancing systemic antitumor immunity in combination with αPD-1 therapy.

In this context, we constructed a multifunctional liposomal nanoplatform, termed LQPO (Liposome-Quantum dot-PFH-Ozone), in which MXene-derived Ti_3_C_2_ quantum dots (QDs) serve as the central “engine” that orchestrates all diagnostic and therapeutic functions. By integrating Ti_3_C_2_ QDs into the lipid bilayer, the system achieves robust NIR-II absorption and high photothermal conversion efficiency, allowing simultaneous deep-tissue PTT and PAI activation under one NIR-II laser irradiation 17-21. The liposomal core encapsulates perfluorohexane (PFH), which acts as a phase-change medium and a reservoir for stably dissolved ozone (O_3_). Upon intravenous administration, LQPO accumulates in tumors *via* the enhanced permeability and retention effect. NIR-II irradiation first activates the Ti_3_C_2_ QDs to generate strong PAI signals for pre-treatment navigation, and simultaneously converts light into localized heat to initiate PTT. The resulting temperature rise triggers acoustic droplet vaporization of PFH, causing liposomal disruption and on-demand release of O_3_ to induce oxidative stress-mediated gas therapy. The generated PFH microbubbles further function as excellent CEUS contrast agents, providing intra-treatment CEUS imaging to visually confirm therapeutic activation and spatial coverage 22. Through this precisely coordinated cascade, LQPO integrates NIR-II-guided PTT with O_3_-based ROS therapy, enabling both dual-modal PAI/CEUS imaging and robust ICD induction. It simultaneously remodels the TME into an immunologically “hot” state to potentiate αPD-1 immunotherapy, offering a comprehensive theranostic strategy for refractory TNBC (Figure 1) 23.

## Materials and Methods

### Material

MXene, hydrofluoric acid (HF), tetrapropylammonium hydroxide (TPAOH), and (3-aminopropyl) triethoxysilane (APTES) were purchased from Aladdin. Lecithin and cholesterol were obtained from Sangon Biotech Co., Ltd. Perfluorohexane (PFH) was purchased from J&K Scientific Ltd. The near-infrared fluorescent dye DiR was supplied by UElandy. The αPD-1 antibody was kindly supplied by Conmed Biosciences Inc. The apoptosis detection kit was obtained from 4A Biotech Co., Ltd. The ATP assay kit, Calcein-AM/Propidium Iodide (PI) live/dead staining kit, 2,7-dichlorodihydrofluorescein diacetate (DCFH-DA), and lactate dehydrogenase (LDH) cytotoxicity assay kit were purchased from Beyotime Biotechnology Co., Ltd. Z-DEVD-FMK and Necrostatin-1 (Nec-1) were purchased from MedChemExpress. The wheat germ agglutinin (WGA) and counterstained with 4’,6-diamidino-2-phenylindole (DAPI) were purchased from Invitrogen. Fetal bovine serum (FBS) was obtained from Gibco Life Technologies. Trypsin-EDTA, penicillin, and streptomycin were purchased from Merck Millipore. Dulbecco’s modified Eagle’s medium (DMEM) and RPMI-1640 medium were obtained from HyClone. Antibodies including LAMP1 (9091T, human), caveolin-1 (3267S), clathrin (4796S), Caspase-3 (9662S), Cleaved Caspase-3 (9664S), PARP (9542S), Cleaved PARP (5625S), CD31 (77699s), and CD3 (26582S) were purchased from Cell Signaling Technology. Antibodies including LAMP1 (ab208943, mouse), β-Actin (ab8226), GSDME (ab215191), HMGB1 (ab79823), Granzyme B (ab255598), HIF-1α (ab228649), CD8α (ab217344), and CD4 (ab183685) were purchased from Abcam.

### Cells and mice

The murine TNBC cell line 4T1 and the human TNBC cell line BT-549 were obtained from stocks maintained in our laboratory, while MDA-MB-468 cells were purchased from the National Collection of Authenticated Cell Cultures (China). All cell lines were cultured in DMEM or RPMI-1640 complete medium supplemented with 10% FBS, 100 U/mL penicillin, and 100 μg/mL streptomycin. Cells were maintained at 37 °C in a humidified incubator with 5% CO_2_.

Female BALB/c mice were purchased from Beijing HFK Bioscience Co., Ltd. (Beijing, China) and housed under specific pathogen-free (SPF) conditions. Animal handling and experimental procedures were approved by the Ethics Committee for Animal Experimentation of Sichuan University. The study was carried out in accordance with the National Institutes of Health Guide for the Care and Use of Laboratory Animals and the Animal Welfare Act.

### Synthesis of LQPO

Preparation of Mxene Ti_3_C_2_ by Etching Al Layer: Briefly, 5 mL of 40% HF was added into a polytetrafluoroethylene container. Then, 500 mg of Ti_3_AlC_2_ was gradually added to the HF solution. The mixture was stirred at room temperature for over 3 days to etch the Al layer, and the reaction was performed in a fume hood. During the initial phase, the container lid was opened for 2 h to release hydrogen gas and then sealed after the reaction stabilized to prevent oxidation. The resulting black precipitate was washed with deionized water by diluting 1 mL of the reaction mixture with 30 mL of deionized water. The washing time depended on the reaction duration in the previous step. The product was washed for 50 min per cycle at 8500 rpm until the pH of the washing solution was below 6. The resulting Ti_3_C_2_ was resuspended in 1 mL of deionized water.

Preparation of Monolayer Mxene: The 1 mL black suspension obtained above was transferred into a 50 mL round-bottom flask. Under stirring, 5 mL of TPAOH was slowly added dropwise. The solution became viscous and resembled petroleum. Nitrogen gas was introduced into the reaction vessel. The solution was sonicated for 10 min and stirred at room temperature overnight. The product was washed three times with deionized water. For long-term storage, the product was washed with ethanol and stored in ethanol under nitrogen protection in a sealed container. The resulting product was collected as monolayer Ti_3_C_2_.

Preparation of Ti_3_C_2_ QDs: The precipitate from the previous step was resuspended in 30 mL of deionized water. The suspension was sonicated in a water bath under N_2_ protection for 6 h and then transferred into a high-pressure fluorine liner. The suspension was heated at 120 °C for 6 h and centrifuged to collect the precipitate.

Oxidation-Protected Ti_3_C_2_@APTES (QDs): The precipitate collected from the high-pressure fluorine-lined vessel was rinsed repeatedly with ethanol and transferred into a round-bottom flask containing 30 mL of ethanol. The flask was evacuated through a three-way valve and subsequently purged with nitrogen. After the suspension was heated to 50 °C in an oil bath, 1 mL of APTES was slowly introduced using a syringe, and the mixture was stirred for 5 min. The reaction temperature was then raised to 80 °C and maintained for 4 h. Once cooled to room temperature, the resulting precipitate was recovered, washed with ethanol, and preserved in ethanol under protected conditions.

Preparation of Lipo@QDs-NH_2_ (LQP): To prepare the lipid film, 15 mg egg yolk lecithin, 5 mg cholesterol, and 1 mg QDs were dissolved in 5 mL chloroform. The solution was placed in a 100 mL pear-shaped flask and evaporated at 40 °C and 60 rpm using a rotary evaporator, during which boiling and bubble formation were avoided. The obtained film was kept under vacuum for 1 h to remove residual chloroform. In parallel, 120 µL PBS, 20 µL Tween-80, and 100 µL PFH were vortexed for 15 min to form a preliminary emulsion. This emulsion was introduced into 10 mL sterile water for hydration, followed by 20 min of water-bath sonication and filtration through a 0.22 µm membrane.

Ozone saturation (LQPO): The step follows the protocol established in our previous work 10. Specifically, ozone was introduced at a rate of 0.5 NL/min for 2 min into 5 mg of Lipo@QDs-NH_2_ dispersed in 1 mL of PBS, resulting in the formation of LQPO.

### LQPO characterization

Transmission electron microscopy (TEM) imaging was conducted using a FEI Talos F200X equipped with a field emission gun operating at 200 kV. Nanoparticle size and Zeta potential were characterized *via* the ZetaView TWIN PMX-220 system. Gas chromatography (Agilent 7890B) was utilized to quantify the PFH content within the nanoparticles. The optical absorption spectra of LQPO were recorded using a Shimadzu UV-3600 UV-*Vis*-NIR spectrophotometer to evaluate its NIR-II absorption characteristics. For thermal imaging, the photothermal response of LQPO under NIR-II laser irradiation was monitored using a FLIR handheld infrared thermal camera (FLIR Systems, USA). The ozone loading and release behavior of LQPO were evaluated as follows: 1 mL of the material was first saturated with ozone and then diluted with 30 mL of ultrapure water. The ozone concentration in the suspension was subsequently monitored at predetermined time intervals using a pen-type ozone detector (Clean Instruments Co., Ltd., Taiwan, China). The O_3_ generation capability of the LQPO nanosystem was determined *via* a decolorization assay employing potassium indigo trisulfonate as a specific probe. In a typical procedure, 10 µL of the LQPO nanosystem was dispersed into 10 mL of an indigo trisulfonate aqueous solution (0.5 mg/mL). Upon NIR-II laser irradiation (1064 nm), the reactive O_3_ molecules induced the stoichiometric cleavage of the indigo chromophore, resulting in a measurable decrease in absorbance at 600 nm. The molar quantity of the generated O_3_ was subsequently calculated based on the 1:1 reaction stoichiometry between O_3_ and indigo trisulfonate. Specifically, the amount of reacted indigo (Δ*n*, in moles) was derived from the absorbance change using the expression Δ*n* = (*A*_blank_ - *A*_LQPO_) × *V* / (ε×*b*), where *A*_blank_ and *A*_LQPO_ represent the absorbance of the probe solution before and after treatment, respectively, *V* denotes the total reaction volume, *b* is the optical path length (1 cm), and ε is the molar absorptivity of potassium indigo trisulfonate (20000 ± 500 L/(mol·cm) at 600 nm). To ensure accuracy, the background interference from the nanosystem itself was subtracted, and all measurements were conducted in triplicate.

### Cellular uptake and* in vivo* biodistribution assessment of LQPO

To evaluate cell-type-dependent internalization, TNBC cell lines were incubated with NileRed-loaded LQPO. Briefly, 5×10^4^ cells were seeded onto 12-well plates containing coverslips. After overnight growth, cells were treated with Nile Red-loaded LQPO (30 μg QDs, 101.2 μg/mL ozone). Following incubation, cells were washed three times with PBS and fixed with 4% paraformaldehyde for 30 min. Flow cytometry was employed to quantify cellular internalization. After treatment with Nile Red-loaded LQPO as described above, cells were harvested and analyzed by flow cytometry at various time points to monitor internalization dynamics. The percentage of Nile Red-positive cells and the mean fluorescence intensity (MFI) were quantified to evaluate the onset and extent of nanoparticle internalization, respectively.

To investigate the cellular uptake route of LQPO, immunofluorescence (IF) staining was performed using representative markers associated with different internalization pathways. Cells were first exposed to Nile Red-loaded LQPO containing 30 μg QDs and 101.2 μg/mL ozone for 6 h. After incubation, the samples were rinsed three times with PBS, followed by labeling of the plasma membrane with 1 μg/mL Alexa Fluor 488-conjugated WGA on ice for 20 s. The cells were subsequently fixed in 4% paraformaldehyde for 20 min and then blocked and permeabilized with PBS containing 10% BSA and 3% Triton X-100 for 1 h at room temperature. Primary antibodies targeting LAMP1, caveolin-1, and clathrin were prepared in 10% BSA-PBS and applied to the samples overnight at 4 °C. On the following day, the slides were incubated with Alexa Fluor 488-conjugated secondary antibodies for 1 h at room temperature, counterstained with DAPI, and mounted for imaging. High-magnification images were captured and analyzed using Fiji/ImageJ software. Colocalization analysis was performed by calculating the Pearson’s correlation coefficient (PCC) between the Nile Red signal and the corresponding endocytic marker signal from representative fluorescence images.

For biodistribution assessment, 5×10^5^ 4T1 cells were subcutaneously implanted into the left dorsal region of BALB/c mice. When tumors reached approximately 200 mm^3^, DiR-labeled LQPO was administered *via* tail vein injection. Fluorescence imaging was conducted *in vivo* at 1, 2, 4, 6, 12, 24 and 48 h post-injection to monitor nanoparticle accumulation and clearance. At 48 h, the mice were euthanized, and the major organs (heart, liver, spleen, lungs, and kidneys) along with tumors were excised for *ex vivo* fluorescence imaging. All fluorescence signals were acquired using a Spectral Instruments *in vivo* imaging system (Spectral Instruments Imaging, USA, λ_ex_ = 748 nm, λ_em_ = 780 nm). The exposure time was fixed at 5 s per frame, and the stage height and illumination arm settings were kept constant throughout all acquisitions to ensure comparability. For each group, three replicate samples were examined. The fluorescence intensity was measured using AMI View software (Spectral Instruments Imaging, USA) to support quantitative analysis of nanoparticle biodistribution.

### Cytotoxicity measurement

Cytotoxicity was evaluated using the methyl thiazolyl tetrazolium (MTT) assay. The 4T1 and BT-549 cell lines were plated at a density of 1500 cells/well in 96-well plates. For the NIR-II laser-treated group, NIR-II laser irradiation (0.5 W/cm^2^) was applied following nanoparticle addition. After an additional 48 hours of incubation, 10 μL of MTT solution (5 mg/mL) was added to each well, and the plates were incubated for 6 hours. Subsequently, 100 μL of SDS-HCl solution was added to dissolve formazan crystals, and the plates were kept overnight at 37 °C. Absorbance at 570 nm was measured using a multifunctional microplate reader.

A clonogenic assay was performed to investigate the long-term effects of this treatment. Cells were seeded into 6-well plates at a density of 5000 cells/well and allowed to adhere overnight. The next day, LQPO (30 μg QDs, 101.2 μg/mL ozone), LQP (30 μg QDs) and LPO (101.2 μg/mL ozone) were applied following the same protocol as the MTT assay. The culture medium was refreshed every two days to ensure optimal growth conditions. After 14 days, the resulting colonies were fixed with 4% paraformaldehyde and stained with 0.05% crystal violet for visualization.

Fluorescence imaging with Calcein-AM and PI was used to distinguish live and dead cells. Cells were seeded in 6-well plates at 1 × 10^4^ cells/well and cultured overnight to permit attachment. Following treatment for 6 h, the cells were labeled with 2 μM Calcein-AM for 30 min under light-protected conditions and then rinsed three times with PBS. Subsequently, 4.5 μM PI was added, and the cells were imaged using an inverted fluorescence microscope (SONY). The percentage of PI-positive cells in live/dead staining images was quantified using ImageJ software by calculating the ratio of PI-positive cells to the total number of cells in each field.

### Pyroptosis determination and rescue assays

Cells were exposed to LQPO (containing 30 μg QDs and 101.2 μg/mL ozone), LQP (30 μg QDs), or LPO (101.2 μg/mL ozone), with or without NIR-II laser irradiation. Following 8 h of incubation, cell death modes were preliminarily analyzed using Annexin V-FITC/PE-PI double staining. Briefly, cells were harvested and rinsed with PBS, followed by resuspension in binding buffer and staining with Annexin V-FITC and PE-PI for subsequent flow cytometry.

LDH release was measured to assess membrane damage associated with pyroptotic cell death. Briefly, culture supernatants were collected after the indicated treatments and centrifuged to eliminate residual cells and debris. The resulting supernatants were incubated with the LDH assay working solution, followed by absorbance measurement using a microplate reader. The LDH release level was normalized to the corresponding control group. LDH release was calculated as a percentage of total LDH release after complete cell lysis using the following formula: (Experimental value - Background control) / (Maximum LDH release control - Background control) × 100%, where background control represented spontaneous LDH from cell cultures, and maximum LDH release control represented complete lysis of all cells.

For rescue experiments, cells were pretreated with Z-DEVD-FMK (20 μM, 1 h) or Nec-1 (30 μM, 1 h) prior to exposure to LQPO treatments with NIR-II irradiation. Following treatment, Annexin V-FITC/PE-PI flow cytometry and LDH release assays were performed as described above to evaluate whether inhibition of caspase-3/GSDME-mediated pyroptosis or necroptosis could alter the cell death phenotype.

For Western blotting, approximately 1 × 10^5^ cells were seeded in 6-well plates and treated under the same conditions. Cells were then lysed using RIPA lysis buffer containing protease and phosphatase inhibitors. Equal amounts of total protein were separated by SDS-PAGE and transferred onto polyvinylidene fluoride membranes. The membranes were blocked and incubated overnight at 4 °C with the following primary antibodies: Caspase-3, Cleaved Caspase-3, PARP, Cleaved PARP, β-Actin, and GSDME. Following the washing steps, HRP-conjugated secondary antibodies were applied to the membranes. The immunoreactive bands were then detected with an enhanced chemiluminescence reagent (Thermo Fisher Scientific, USA).

### ICD evaluation

Cells were seeded onto pre-coated slides within wells and subjected to various treatments. Afterward, the cells were fixed using cold methanol. Fixed cells were then incubated with an HMGB1 primary antibody at room temperature for 1 hour. Following primary antibody incubation, Alexa Fluor 488-conjugated antibody was applied after thorough PBS washing, and the cells were incubated for an additional hour in the dark at room temperature. After the last wash with PBS, nuclei were counterstained and the samples were mounted using DAPI Fluoromount-G, followed by image acquisition under a confocal microscope (STELLARIS 5, Leica Microsystems, Germany). Extracellular ATP secretion was measured using an ATP assay kit. Specifically, cells were plated into 6-well plates at a density of 1×10^5^ cells per well. After a 2-h exposure to different treatments, the supernatants were carefully collected for analysis. ATP levels were quantified according to the manufacturer’s instructions.

### CEUS and PA imaging

In the *in vitro* ultrasound imaging assay, LQPO samples with QD concentrations of 0 to 30 μg/mL were embedded in a 1% agarose gel model with PCR tubes and exposed to NIR-II irradiation at 0.5 W/cm^2^. B-mode and CEUS images were then recorded with a Philips iU22 ultrasound system. For the *in vivo* study, TNBC-bearing mice received LQPO through tail vein injection at a dose of 1.2 mg/kg QDs and 0.5 mg/kg ozone. At 1 h post-injection, tumors were examined in both B-mode and CEUS mode using the ultrasound system. The imaging of the tumor observed after NIR-II irradiation (0.5 W/cm^2^, 10 min).

NIR-II PAI all performed on Vevo LAZR-Xsystem (VisualSonics Inc. New York, NY). For evaluating PA performance of LQPO *in vitro*, aqueous solution with gradient increasing concentration of the sample (QD: 0-30 μg/mL) employed. The system setting parameters were as follows: frequency = 21 MHz, wavelength range = 1200-2000 nm, PA gain = 50 dB, gain = 15 dB depth/width = 10.00/14.08 mm.

For *in vivo* multispectral PA imaging, 4T1 tumor-bearing mice were intravenously injected with LQPO (1.2 mg/kg QDs, 0.5 mg/kg ozone), LQP (1.2 mg/kg QDs), or LPO (0.5 mg/kg ozone). Tumors were scanned using the PA-Mode 3D (Multiwavelength) and PA-Mode 3D (Oxy-Hemo) settings to reconstruct 3D PA images and evaluate oxygenation-related parameters. To distinguish the exogenous LQPO signal from endogenous oxygenated hemoglobin (OxyHb) and deoxygenated hemoglobin (DeoxyHb), spectral unmixing as performed using the Linear Least Squares Unmixing algorithm in Vevo Lab software. Pre-installed reference spectra for OxyHb and DeoxyHb were used for endogenous chromophore separation, while the characteristic absorption profile of LQPO determined by *in vitro* multispectral scanning, with a local absorption maximum identified at 1265 nm (Figure S4A). Unmixing based on multiwavelength fitting at the pixel level, allowing estimation of the relative contribution of each chromophore despite the broad NIR-II absorption of Ti_3_C_2_ QDs.

The system setting parameters were as follows: frequency = 40 MHz; wavelength range = 1200-2000 nm; PA gain = 50 dB; gain = 22 dB depth/width = 10.00/14.08 mm; wavelength (LQPO) = 1265 nm; 3D step size = 0.13 mm; scanning time: single-spectral PA imaging (about 1 min) and multispectral PA imaging (about 6 min); scanning area: ~500 mm^2^; pulse repetition rate: 20 Hz; safe energy: 20 mJ/cm^2^. Quantitative analysis of OxyHb, DeoxyHb, Total Hemoglobin (HbT), and Oxygen Saturation (sO_2_) performed using the Region of Interest (ROI) analysis tool in Vevo Lab software.

### Experimental protocol *in vivo*

All animal experiments and related protocols were approved by the Administration Committee of Experimental Animals at West China Hospital, Sichuan University. The mice were housed under standard conditions with unrestricted access to food and water. To assess the therapeutic efficacy of NIR-II-triggered LQPO treatment, a bilateral 4T1 tumor model was established in BALB/c mice by subcutaneously inoculating 5 × 10^5^ 4T1 cells into both dorsal flanks. When the tumors reached approximately 100 mm^3^, the mice were randomly allocated to different treatment groups. In this model, only the tumor on the left dorsal side, defined as the primary tumor, received NIR-II laser irradiation, whereas the contralateral tumor was regarded as the abscopal tumor and was not exposed to laser irradiation, enabling assessment of systemic anti-tumor responses. Mice were treated via tail vein injection every three days as follows: (1) PBS (blank), (2) NIR-II irradiation alone (0.5 W/cm^2^, 10 min), (3) LQPO (1.2 mg/kg QDs, 0.5 mg/kg ozone), (4) LQPO + NIR-II, (5) LQP (1.2 mg/kg QDs), (6) LQP + NIR-II, (7) LPO (0.5 mg/kg ozone), and (8) LPO + NIR-II. Tumor growth was followed every three days using a vernier caliper, and tumor volume was determined using the formula: Volume = length × width^2^ / 2. On day 15, the mice were euthanized, and tumor tissues were excised for subsequent analyses.

To assess the immunotherapeutic potential of NIR-II-activated LQPO, mice bearing bilateral 4T1 tumors (~100 mm^3^) were randomly assigned to four groups: (1) Blank, (2) LQPO+NIR-II (1.2 mg/kg QDs, 0.5 mg/kg ozone; 0.5 W/cm^2^, 10 min), (3) αPD-1 (3 mg/kg, intraperitoneally), and (4) LQPO+NIR-II+αPD-1. Only the left (primary) tumor receives NIR-II irradiation, while the contralateral (abscopal) tumor remains unirradiated to evaluate systemic effects. LQPO was administered *via* tail vein injection, and αPD-1 antibodies were given intraperitoneally at day 1 and day 9. Tumor growth was monitored and calculated every 3 days according to the procedure described above. On day 15, mice were euthanized, and both primary and abscopal tumors were collected for histological and immunological evaluations.

### Immunohistochemistry and immunofluorescence staining

Tumor tissues were fixed in 10% formalin, paraffin-embedded, and sectioned at 4 μm thickness. Hematoxylin and eosin (H&E) staining was performed at the Laboratory of Pathology, West China Hospital, Sichuan University. For immunohistochemistry (IHC) and immunofluorescence (IF) analyses, paraffin sections were first deparaffinized and rehydrated through graded ethanol.

For IHC staining, endogenous peroxidase activity was blocked by incubation with peroxidase blocking buffer for 15 min, followed by blocking with 5% BSA in PBS at room temperature for 1 h. The tissue sections were probed overnight at 4 °C with primary antibodies targeting cleaved GSDME, HMGB1, CD31, Granzyme B, and HIF-1α. After rinsing in PBS, species-specific HRP-conjugated secondary antibodies were applied, and DAB was used for chromogenic signal detection. The nuclei were subsequently stained with hematoxylin. The samples were then dehydrated stepwise in increasing concentrations of ethanol, cleared, coverslipped, and imaged. The staining intensity of cleaved GSDME and HMGB1 was quantified using Image Pro Plus 6.0 software by calculating the integrated optical density (IOD) relative to the stained area. For Granzyme B-stained sections, the number of positive cells in each field was quantified. For HIF-1α-stained sections, the percentage of positive cells in each field was quantified as an indicator of intratumoral hypoxia. For CD31-stained sections, vascular morphology was further analyzed by measuring the minimal vessel diameter and luminal area to evaluate treatment-induced vascular remodeling. These parameters were quantified using ImageJ.

For immunofluorescence staining, deparaffinized sections were blocked in 5% BSA-PBS for 1 h and then probed overnight at 4 °C with primary antibodies targeting CD3, CD8α, and CD4. After primary antibody incubation, the sections were labeled with Alexa Fluor 488- or Alexa Fluor 647-conjugated secondary antibodies for 1 h at room temperature in the dark. Nuclear staining was performed with DAPI, and the samples were mounted using antifade Fluoromount-G for subsequent fluorescence imaging.

For oxidative-stress-associated fluorescence staining, freshly collected tumor tissues were embedded in optimal cutting temperature (OCT) compound and cryosectioned into 10 μm frozen sections. The sections were incubated with dihydroethidium (DHE) in the dark according to the manufacturer’s instructions, washed with PBS, and counterstained with DAPI. Fluorescence images were acquired using a fluorescence microscope or confocal laser scanning microscope with an excitation wavelength of 535 nm and an emission wavelength of 610 nm. All images were captured under identical acquisition settings for different groups.

### Hemolysis assay

A hemolysis assay was performed to evaluate the blood compatibility of the nanoplatform. Fresh anticoagulated blood was collected, and red blood cells were isolated by centrifugation, washed with PBS, and resuspended to obtain a red blood cell suspension. The red blood cell suspension was then incubated with the indicated formulations for 0, 1, 2, and 6 h. PBS and deionized water were used as the negative and positive controls, respectively. After incubation, the samples were allowed to stand for natural sedimentation, and representative photographs were recorded to evaluate hemolysis qualitatively based on the color of the supernatant and the sedimentation status of red blood cells.

### Statistical analysis

All quantitative data were presented as the mean ± standard deviation (SD) from at least three independent experiments. Statistical analyses were performed using GraphPad Prism software. For comparisons among more than two groups at a single time point, including *in vitro* cell viability, PI-positive population, LDH release, terminal tumor weights, and quantitative IHC/IF measurements, one-way analysis of variance (ANOVA) followed by Tukey’s post hoc test was used. For longitudinal or time-dependent datasets, including *in vivo* tumor growth curves, dynamic photoacoustic parameters (e.g., LQPO signal, OxyHb, HbT, and sO_2_), and *in vitro* cellular uptake kinetics, two-way ANOVA was applied. A *P* value of less than 0.05 was considered statistically significant. Statistical significance was indicated as *P < 0.05, **P < 0.01, ***P < 0.001, and ****P < 0.0001.

## Results

### Construction and characterization of LQPO

**Figure 2:**
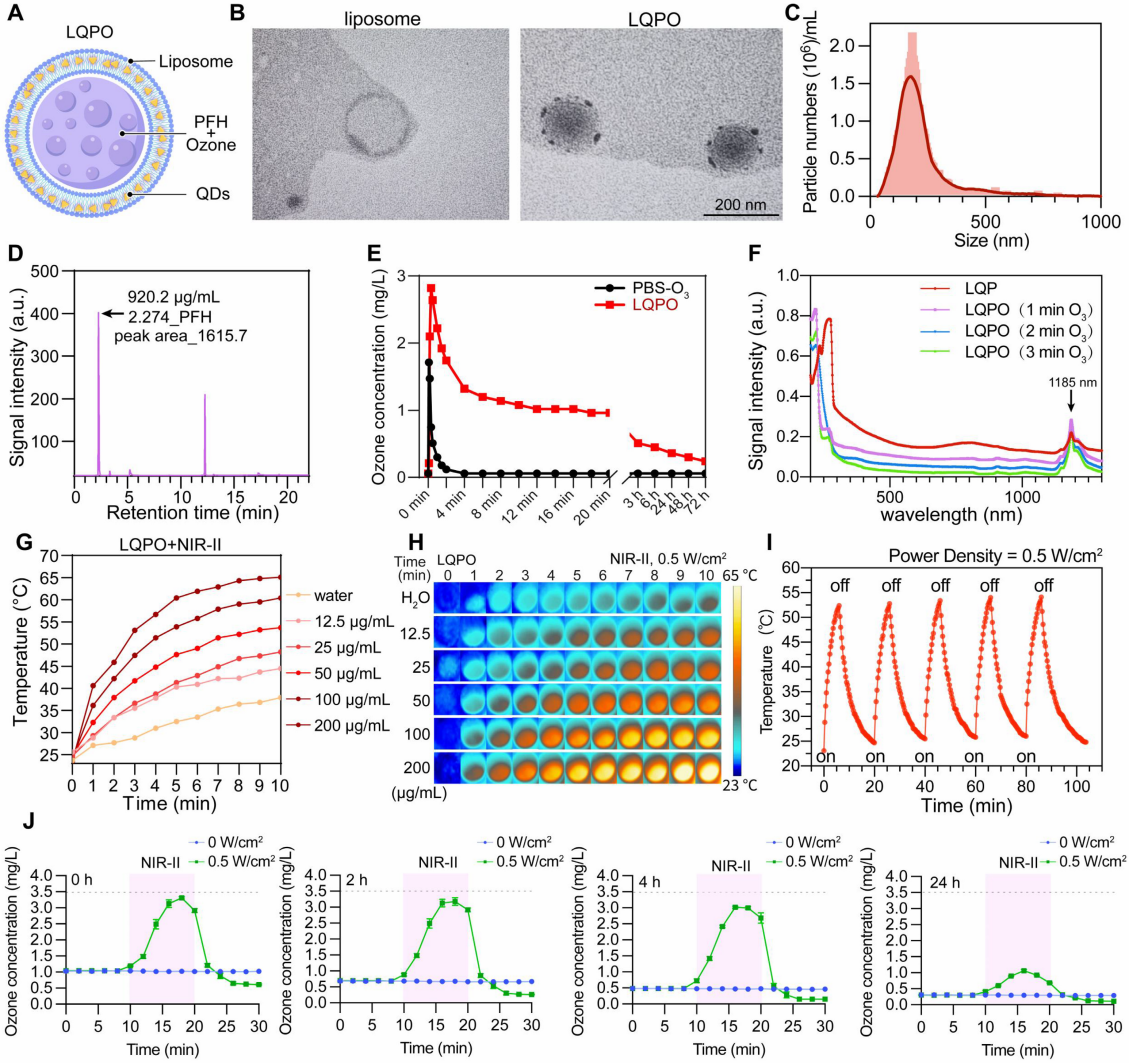
** Structural characterization and photothermal-ozone properties of the LQPO nanoplatform.** (A) Schematic illustration of the NIR-II light-triggered liposome-quantum dot-PFH-ozone (LQPO) nanoplatform. (B) Transmission electron microscopy (TEM) images of blank liposomes (left) and LQPO (right), where dense black dots in the bilayer membrane indicate the successful incorporation of Ti_3_C_2_ QDs. Scale bar = 200 nm. (C) Hydrodynamic size distribution of LQPO with an average diameter of 200 nm. (D) Gas chromatography analysis confirming PFH encapsulation with a quantified content of 920.2 μg/mL. (E) Ozone retention profiles of LQPO and ozone-saturated PBS. Data are presented as mean ± standard deviation (SD) (n = 3). (F) Ultraviolet-visible-near-infrared (UV-*vis*-NIR) absorption spectra of Ti_3_C_2_ QDs and LQPO after different ozone-loading times. (G) Temperature elevation curves of LQPO at different concentrations under NIR-II laser irradiation (0.5 W/cm^2^, 10 min). (H) Infrared thermal images corresponding to (G), showing concentration-dependent photothermal heating. (I) Photothermal stability of LQPO under repeated on/off laser irradiation cycles. (J) NIR-II-triggered ozone release profiles of LQPO after different pre-incubation times (0, 2, 4, and 24 h), with or without laser irradiation (0.5 W/cm^2^). Data are presented as mean ± standard deviation SD (n = 3).

The photothermal NIR-II laser-controlled ozone release nanosystem LQPO was designed and synthesized as outlined in Figure 1A. Briefly, Ti_3_AlC_2_ was etched with hydrofluoric acid to remove the Al layers, followed by intercalation with TPAOH and ultrasonic exfoliation to obtain monolayer Ti_3_C_2_ nanosheets. These nanosheets were further treated under hydrothermal conditions to yield Ti_3_C_2_ QDs, which were subsequently modified with APTES to form a protective oxide layer against ozone-induced degradation and to render the QDs hydrophobic, thereby facilitating their stable incorporation into the lipid bilayer. The APTES-modified QDs were then embedded within liposomes composed of lecithin and cholesterol, while the liquid PFH core was pre-loaded with ozone as a therapeutic gas reservoir. The final nanostructure, termed LQPO, thus integrated Ti_3_C_2_ QDs in the liposomal bilayer with ozone-saturated PFH encapsulated in the core (Figure 2A). This preferential loading leverages the unique fluorous phase effect and high free volume of perfluorocarbons, allowing the PFH core to act as a high-capacity reservoir that efficiently stabilizes non-polar O_3_ by shielding it from the rapid degradation typical of aqueous environments 24. The morphology and structural features of the prepared liposomes and LQPO were first examined by TEM. As shown in Figure 2B, the blank liposomes exhibited a typical spherical vesicular structure with clear bilayer membranes, whereas the LQPO nanoparticles displayed numerous dark dots embedded in the lipid bilayer, indicating the successful loading of Ti_3_C_2_ QDs within the liposomal membrane. Dynamic light scattering analysis further confirmed that LQPO exhibited a uniform size distribution with an average hydrodynamic diameter of approximately 200 nm (Figure 2C), with a Zeta potential of -25 mV. Furthermore, longitudinal monitoring in PBS and 10% FBS revealed that LQPO maintained a stable hydrodynamic size and Zeta potential over 72 h, indicating excellent colloidal stability and potential for structural integrity during systemic circulation (Figure S1A-B). Gas chromatography demonstrated the efficient encapsulation of PFH within the liposomal core, with a quantified PFH content of 920.2 μg/mL (Figure 2D). To evaluate the ozone-loading capacity, equal volumes of LQPO and ozone-saturated PBS were dispersed in water, and the dissolved ozone concentration was monitored. Compared with the PBS group, which rapidly lost ozone within 4 min, LQPO exhibited a markedly higher ozone retention, with an initial concentration of 2.9 mg/L and sustained levels of ~1 mg/L at 20 min, followed by a slower long-term release (Figure 2E). To further quantify the ozone payload, the loading efficiency (LE) of O_3_ in LQPO was determined using the potassium indigo trisulfonate spectrophotometric method under NIR-II-triggered release conditions. The LE was measured to be 0.4% (4 μg O_3_ per mg LQPO), indicating that the PFH-liposomal core can stably accommodate a quantifiable therapeutic dose of ozone. Next, we investigated the optical absorption properties of LQPO. As shown in Figure 2F, the characteristic NIR absorption peak of Ti_3_C_2_ QDs at 1185 nm was well preserved even after ozone encapsulation, indicating that APTES modification effectively prevented oxidative degradation and maintained the intrinsic photothermal properties of the QDs. To establish the intrinsic photothermal conversion capability of Ti_3_C_2_ QDs, aqueous suspensions of pure QDs at different concentrations were irradiated with an NIR-II laser (0.5 W/cm^2^). A clear concentration-dependent temperature increase was observed (Figure S1C-D), with the temperature reaching approximately 72 °C at 200 μg/mL after 10 min of irradiation, confirming the high photothermal efficiency of Ti_3_C_2_ QDs. Under identical irradiation conditions, the assembled LQPO nanosystem, in which Ti_3_C_2_ QDs were embedded within the lipid bilayer, also exhibited pronounced concentration-dependent photothermal heating (Figure 2G-H), achieving temperatures above 65 °C at 200 μg/mL after 10 min. Although lipid encapsulation slightly reduced the peak temperature, LQPO retained robust photothermal responsiveness after integration into the lipid-based architecture. Importantly, LQPO also exhibited excellent photothermal stability during five consecutive on/off laser cycles (Figure 2I), showing negligible loss of heating efficiency. Together, these results demonstrate that Ti_3_C_2_ QDs act as a highly efficient and stable photothermal transducer within the LQPO architecture, endowing the nanosystem with robust, durable, and controllable NIR-II photothermal conversion performance, which provides a reliable basis for achieving precise and on-demand therapeutic activation. To further evaluate the NIR-II-triggered ozone release kinetics, LQPO suspensions were irradiated at varying laser power densities. As shown in Figure S1E, the ozone concentration in the LQPO suspension exhibited a rapid, power-dependent burst immediately upon irradiation, demonstrating that the photothermal effect of Ti_3_C_2_ QDs effectively triggers the release of encapsulated ozone from the PFH-loaded core. Notably, this release capability remained robust even after 24 h of incubation (Figure 2J), confirming the high structural stability and controllable activation of the nanoplatform. This on-demand release profile ensures that the therapeutic gas was delivered precisely during the laser-on period.

### Tumor inhibition of LQPO *in vitro*

**Figure 3:**
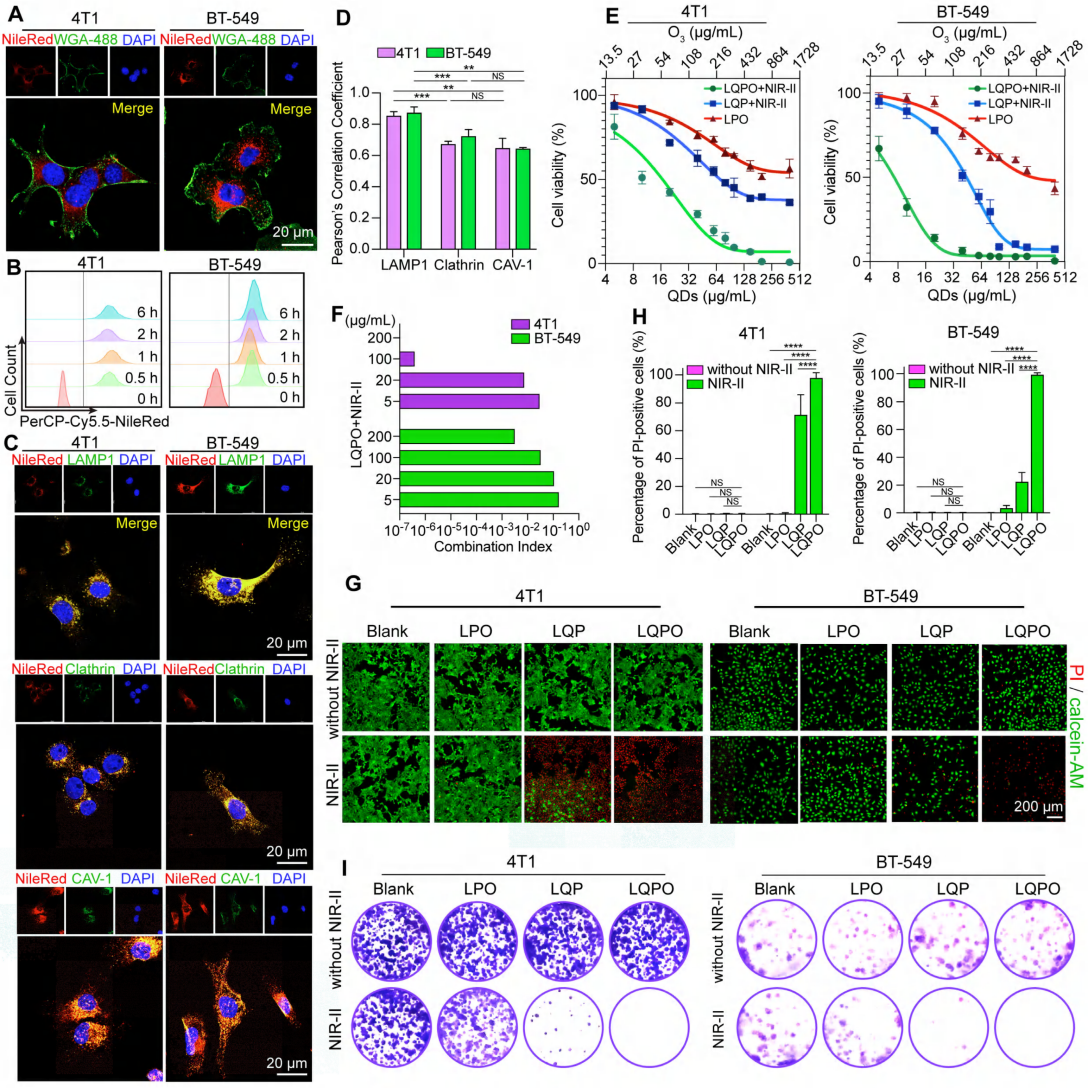
** Cellular uptake and *in vitro* therapeutic efficacy of LQPO.** (A) Confocal fluorescence images of 4T1 and BT-549 cells showing intracellular trafficking of Nile Red-labeled LQPO (red). Cell membranes were stained with WGA-488 (green). Scale bars = 20 μm. (B) Flow cytometry analysis of time-dependent uptake of LQPO (0.5-6 h) in 4T1 and BT-549 cells. (C-D) Confocal fluorescence images and Pearson’s correlation coefficient (PCC) analysis of the colocalization of LQPO with lysosome-associated membrane protein 1 (LAMP1), clathrin, and caveolin-1 (CAV-1) in 4T1 and BT-549 cells. Scale bars = 20 μm. Data are presented as mean ± standard deviation (SD) (n = 4). (E) Cell viability of 4T1 and BT-549 cells treated with LPO (ozone only), LQP+NIR-II (photothermal only), or LQPO+NIR-II (combined therapy) at increasing quantum dot concentrations, measured by methyl thiazolyl tetrazolium (MTT) assay. Data are presented as mean ± standard deviation (SD) (n = 6). (F) Combination Index (CI) values of LQPO+NIR-II in 4T1 and BT-549 cells, showing strong synergism (CI<1) across multiple concentrations. (G) Live/dead staining of 4T1 and BT-549 cells after the indicated treatments with or without NIR-II irradiation. Green fluorescence indicates calcein acetoxymethyl ester (calcein-AM)-positive live cells, and red fluorescence indicates propidium iodide (PI)-positive dead cells. Scale bar = 200 μm. (H) Quantitative analysis of PI-positive cells in 4T1 and BT-549 cells after the indicated treatments. Data are presented as mean ± standard deviation (SD) (n = 4). (I) Clonogenic assays of 4T1 and BT-549 cells after various treatments with or without NIR-II irradiation. Statistical significance is indicated as *P < 0.05, **P < 0.01, ***P < 0.001, and ****P < 0.0001.

To investigate the cellular internalization behavior of LQPO, fluorescence colocalization experiments were first performed in TNBC cell lines. NileRed-labeled particles were efficiently internalized by 4T1, BT-549, and MDA-MB-468 cells after 2 h of incubation (Figure 3A, Figure S2A). To quantitatively assess the uptake kinetics, flow cytometry was performed at different time points. As shown in Figure 3B and Figure S2B, both 4T1 and BT-549 cells became rapidly NileRed-positive within 0.5 h, indicating fast initial internalization of LQPO, while the MFI continued to increase over time, reflecting progressive intracellular accumulation. Notably, 4T1 cells showed consistently higher MFI values than BT-549 cells, indicating a higher uptake efficiency. This difference may be related, at least in part, to intrinsic biological differences between the two cell lines, including their metabolic activity, endocytic capacity, and cellular heterogeneity 25 (Figure S2B). To further investigate the uptake behavior of LQPO, NileRed-loaded LQPO particles were separately co-visualized with LAMP1, clathrin, and caveolin-1 (CAV-1). LAMP1 was used to indicate lysosomal localization, whereas clathrin and CAV-1 were included to assess possible contributions from clathrin-mediated and caveolin-associated internalization pathways, respectively. As shown in Figure 3C-D, LQPO showed strong colocalization with LAMP1, with a high PCC, indicating that the majority of internalized nanoparticles were trafficked into lysosomal compartments. In addition, moderate but significant colocalization with clathrin and CAV-1 was observed, with corresponding PCC values supporting the involvement of both clathrin-mediated and caveolae-mediated endocytosis pathways. These results demonstrate that LQPO enters cells through multiple endocytic routes, followed by predominant lysosomal trafficking, which is consistent with the intracellular processing pathway of lipid-based nanocarriers.

We next assessed the cytotoxic performance of LQPO under different treatment conditions. As shown in Figure 3E, both LPO (ozone only) and LQP+NIR-II (photothermal only) displayed moderate dose-dependent cytotoxicity in 4T1 and BT-549 cells. In contrast, the LQPO+NIR-II group induced a markedly greater reduction in cell viability, with a half-maximal inhibitory concentration (IC_50_) significantly lower than that of any single-modality treatment. This superior therapeutic effect indicates a potent synergism between photothermal ablation and ozone-mediated oxidative stress. To quantitatively verify this interaction, the Combination Index (CI) was calculated based on the Chou-Talalay method using the *in vitro* cytotoxicity data (Figure 3F). Across multiple concentration levels, the CI values were consistently lower than 1, including at low-to-intermediate dose ranges, demonstrating a robust synergistic interaction between NIR-II photothermal therapy and ozone gas therapy within the LQPO platform.

This effect was also evident in the live/dead staining images (Figure 3G, Figure S2C). In groups without NIR-II exposure, green fluorescence was dominant, suggesting that most cells remained viable as indicated by calcein-AM staining. Upon NIR-II exposure, the LQPO group showed extensive red PI fluorescence, indicating widespread cell death, whereas the LPO and LQP groups induced only partial cell killing. Quantitative analysis of PI-positive cells (Figure 3H, Figure S2D) revealed that the LQPO+NIR-II group reached nearly 100% cell death in both cell lines, significantly exceeding all control groups, further confirming the potent synergistic cytotoxicity of the combined therapy. These findings were further validated by clonogenic assays (Figure 3I, Figure S2E). After 14 days of culture, colonies were abundant in the control and single-modality groups, whereas LQPO+NIR-II almost completely abolished colony formation in 4T1, BT-549, and MDA-MB-468 cells.

Together, these results demonstrate that LQPO can be efficiently internalized by tumor cells *via* multiple endocytic routes, accumulate in lysosomes, and exert potent cytotoxicity through a synergistic photothermal-ozone mechanism, ultimately leading to robust suppression of clonogenic survival.

### LQPO induces pyroptosis and immunogenic cell death in tumor cells

**Figure 4:**
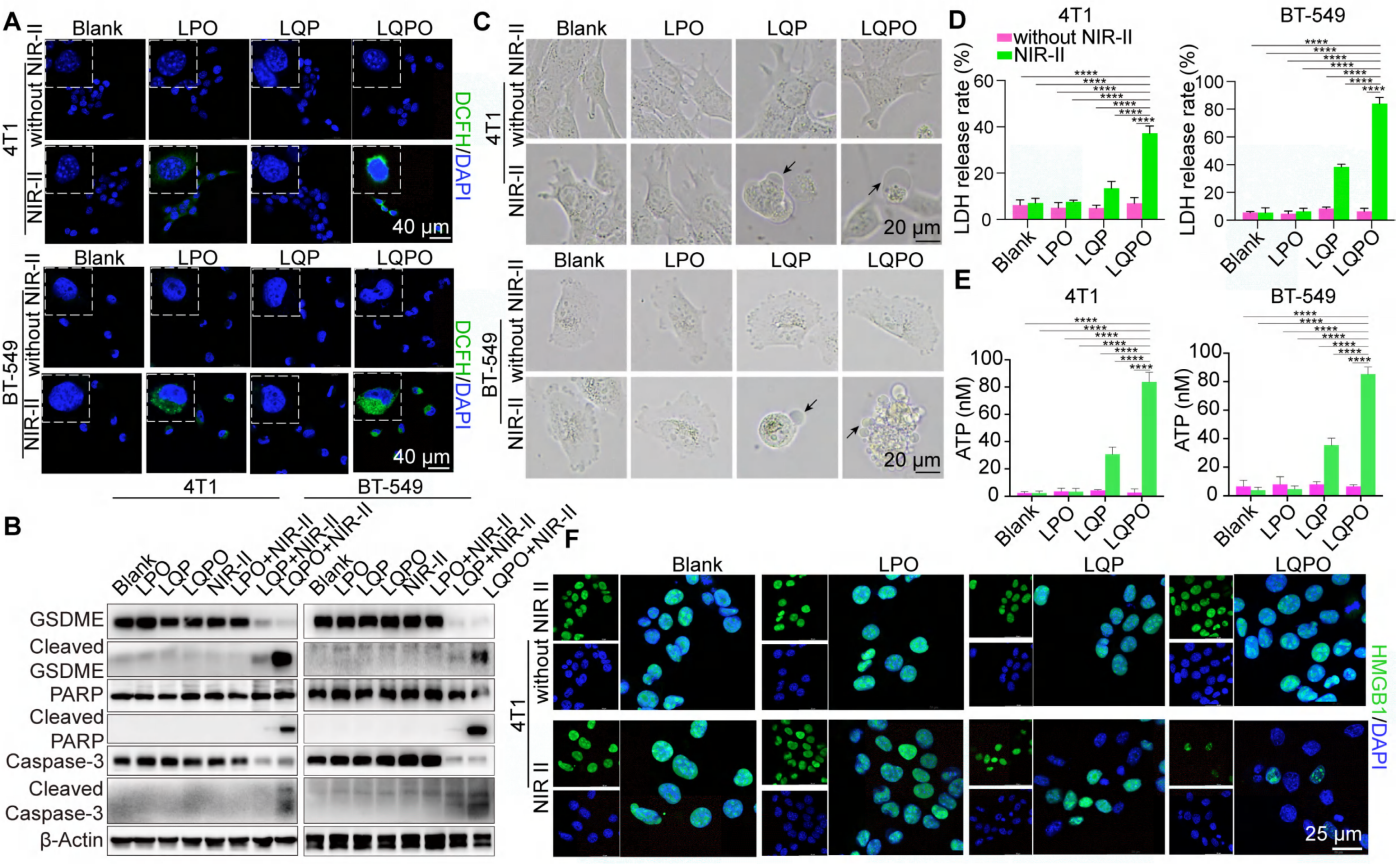
** LQPO induces GSDME-dependent pyroptosis and immunogenic cell death in tumor cells.** (A) Confocal fluorescence images of reactive oxygen species (ROS) generation in 4T1 and BT-549 cells detected by 2’,7’-dichlorodihydrofluorescein diacetate (DCFH-DA) staining after different treatments with or without NIR-II irradiation. Scale bars = 40 μm. (B) Western blot analysis of cell death-related proteins, including gasdermin E (GSDME), cleaved GSDME, poly (ADP-ribose) polymerase (PARP), cleaved PARP, caspase-3, and cleaved caspase-3. β-Actin is used as a loading control. (C) Representative bright-field images of 4T1 and BT-549 cells after different treatment. Black arrows indicate representative cells showing swelling and ballooning morphology consistent with lytic cell death. Scale bar = 20 μm. (D) Lactate dehydrogenase (LDH) release rates in 4T1 and BT-549 cells after the indicated treatments, showing membrane-disruptive lytic cell death. Data are presented as mean ± standard deviation (SD) (n = 3). (E) Extracellular adenosine triphosphate (ATP) release measured in cell supernatants. Data are presented as mean ± standard deviation (SD) (n = 3). (F) Immunofluorescence staining of high mobility group box (HMGB1) in 4T1 cells. Scale bar = 25 μm. Statistical significance is indicated as *P < 0.05, **P < 0.01, ***P < 0.001, and ****P < 0.0001.

To elucidate the mechanism underlying the potent cytotoxicity of LQPO, we first examined the intracellular generation of ROS using DCFH-DA staining (Figure 4A, Figure S3A). Cells treated with LQPO under NIR-II irradiation exhibited strong green fluorescence, indicating massive ROS accumulation, whereas only weak signals were detected in the control or single-modality groups. We then assessed whether the observed cell death was mediated by typical apoptosis using Annexin V/PI staining (Figure S3B). Classically, an apoptotic death is characterized by a predominance of Annexin V^+^/PI^-^ cells (early apoptosis) and a subsequent increase in Annexin V^+^/PI^+^ cells (late apoptosis/necrosis). However, in the LQPO+NIR-II group, a substantial proportion of cells appeared in the Annexin V^+^/PI^+^ (Q2) and more notably, a significant population was Annexin V^-^/PI^+^ (Q1), indicating extensive membrane rupture that implicates lytic programmed cell death (e.g., pyroptosis or necroptosis) over typical apoptosis rather than typical apoptotic progression, thereby implicating non-canonical apoptosis pathways. To further clarify the cell death form, apoptosis- and pyroptosis-related protein markers were analyzed by western blot (Figure 4B). In both 4T1 and BT-549 cells, LQPO+NIR-II treatment robustly activated caspase-3 and induced pronounced cleavage of GSDME, accompanied by a marked reduction of full-length GSDME, indicating extensive GSDME pore formation. Notably, LQP+NIR-II (photothermal treatment) also led to detectable caspase-3 activation and partial GSDME cleavage, consistent with previous reports that intense photothermal stress can initiate GSDME-dependent secondary pyroptosis 26, 27. However, the extent of GSDME processing was substantially enhanced in the LQPO+NIR-II group, suggesting that ozone-mediated oxidative stress amplifies and redirects photothermal-induced apoptotic signaling toward a dominant GSDME-dependent pyroptotic cell death program. Although cleaved PARP was observed, the preferential activation of the caspase-3-GSDME axis supported pyroptosis as the primary death form induced by the combined treatment. Bright-field imaging further revealed prominent cell swelling and large bubble-like membrane protrusions in the LQPO+NIR-II group, providing morphological evidence consistent with lytic pyroptotic cell death (Figure 4C, Figure S3C). The membrane-disruptive nature of cell death was further supported by the LDH release assay, in which the LQPO+NIR-II group exhibited significantly higher LDH leakage than the single-modality and control groups (Figure 4D, Figure S3D). To further distinguish whether LQPO+NIR-II inducing pyroptosis or necrosis, we next performed rescue experiments to define the cell death program using Z-DEVD-FMK, which blocks the caspase-3/GSDME axis in this context, and Nec-1, a necroptosis inhibitor. Flow cytometry analysis (Figure S3E) showed that Z-DEVD-FMK markedly rescued cells from death, with the live cell population (Q4) restored to over 90% in both cell lines. In contrast, Nec-1 treatment had little effect on the population distribution, with cells remaining predominantly in the Annexin V^+^/PI^+^ and Annexin V^-^/PI^+^ quadrants. Consistently, the elevated LDH release was significantly reduced by Z-DEVD-FMK but remained largely unchanged after Nec-1 treatment (Figure S3F). In addition, the formation of membrane bubbles in LQPO+NIR-II group was markedly attenuated by Z-DEVD-FMK, whereas Nec-1 treatment failed to prevent this phenotype (Figure S3G). Together, these findings strongly support that the observed lytic cell death was predominantly mediated through the caspase-3/GSDME axis rather than necroptosis.

Given that pyroptosis can initiate ICD, we next measured the release of ICD-associated signals. ATP release into the culture supernatant was markedly increased in the LQPO+NIR-II group relative to the control and single-treatment groups (Figure 4E, Figure S3H). Crucially, this surge in extracellular ATP was markedly abolished by the addition of Z-DEVD-FMK, further substantiating that the induction of ICD is a direct consequence of the Caspase-3-mediated pyroptotic pathway rather than random cell lysis (Figure S3I). In parallel, IF staining revealed pronounced extracellular release and nuclear depletion of HMGB1 (Figure 4F, Figure S3J). Together, these results demonstrate that LQPO not only induces pyroptosis but also provokes ICD, which are key drivers for remodeling the tumor immune microenvironment, thereby laying the foundation for potential synergy with immune checkpoint blockade therapy.

### Multimodal imaging and oxygenation modulation of LQPO

To demonstrate the deep-tissue imaging advantage of the NIR-II window, we performed a comparative penetration study using a chicken breast tissue model (Figure S4A-C). While the PA signal in the NIR-I window (750 nm) was nearly undetectable at depths greater than 11 mm, the LQPO signal in the NIR-II window (1265 nm) remained clearly detectable at a depth of approximately 15 mm. These results support the improved penetration capability of the LQPO platform in the NIR-II window.

**Figure 5:**
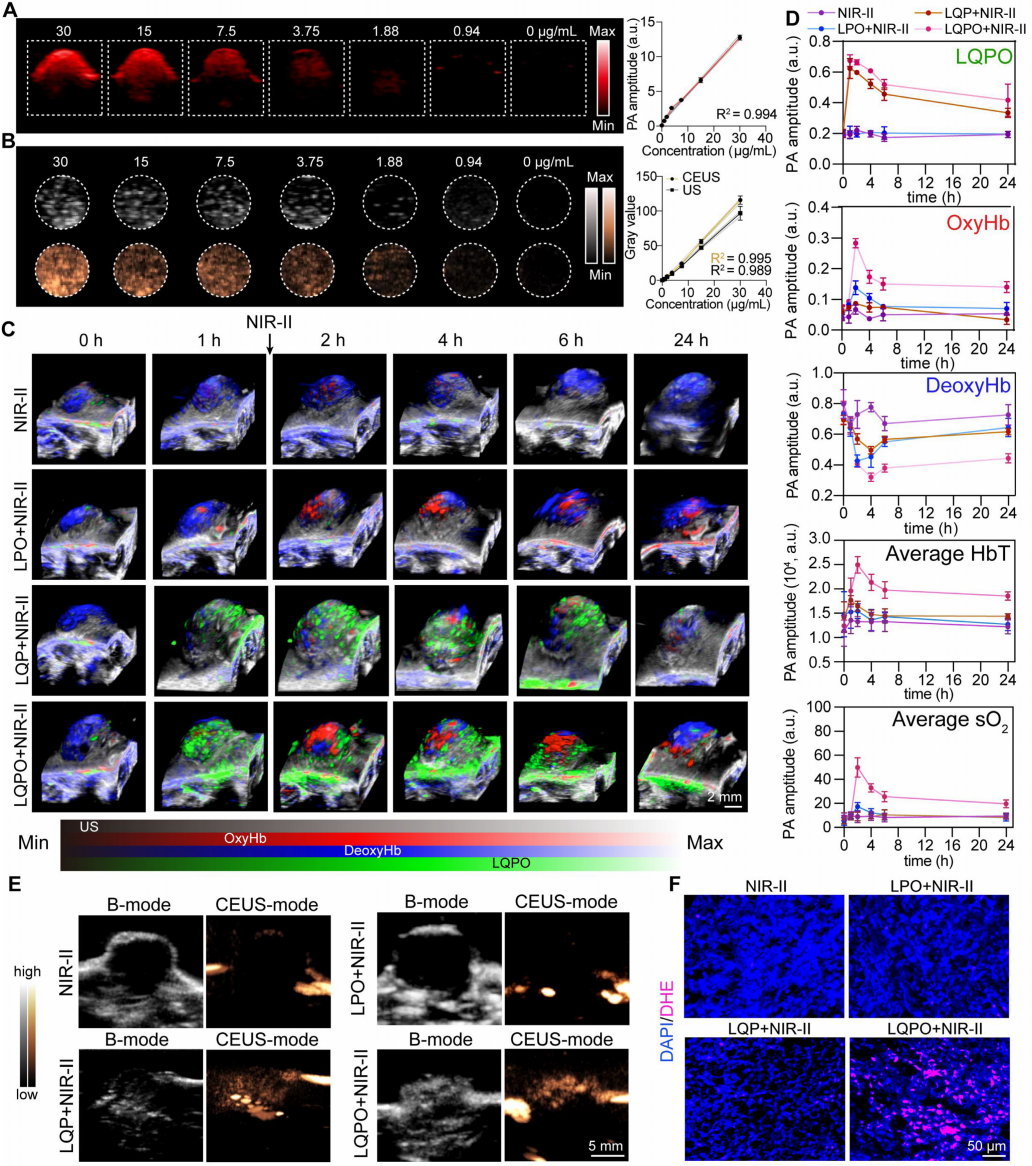
** Multimodal imaging performance and oxygenation modulation of LQPO.** (A) *In vitro* photoacoustic imaging (PAI) of LQPO at different concentrations and the corresponding quantitative analysis. Data are presented as mean ± standard deviation (SD) (n = 3). (B) *In vitro* ultrasound (US) and contrast-enhanced ultrasound (CEUS) imaging of LQPO at different concentrations, with signal intensity increasing in a concentration-dependent manner; right panel: quantification of gray values. Data are presented as mean ± SD (n = 3). (C) Three-dimensional (3D) reconstructed PAI of tumor-bearing mice after intravenous injection of LQPO, followed by NIR-II laser irradiation at 1 h. Images show spatial colocalization of LQPO (QD signal, green), oxygenated hemoglobin (OxyHb, red), and deoxygenated hemoglobin (DeoxyHb, blue). (D) Quantitative analysis of PA signals at the tumor site, including LQPO (green), OxyHb (red), DeoxyHb (blue), Average hemoglobin (HbT), and Average oxygen saturation (sO_2_), at the indicated time points. Data are presented as mean ± SD (n = 3). (E) Representative B-mode and CEUS-mode images of tumors after different treatments. (F) Fluorescence images of reactive oxygen species (ROS) generation in different tumor groups, detected by dihydroethidium (DHE) staining. Scale bars = 50 μm.

To verify the multimodal imaging capability of LQPO, we first conducted phantom experiments for PAI. PAI revealed a strong concentration-dependent signal intensity (Figure 5A), with PA amplitude increasing linearly across the tested concentration range (R^2^ = 0.994), demonstrating its excellent suitability as a PA contrast agent. Based on the calibration curve, the detection limit (LOD) of LQPO was calculated to be 0.038 μg/mL according to the formula LOD = 3σ/k, where σ represents the standard deviation of the blank signal and k is the slope of the linear fit. This sensitivity was within a highly competitive range compared with representative gold nanorod-based PA contrast agents reported in the literature 28, 29. Direct cross-study comparison should be interpreted cautiously, as reported LODs are highly dependent on nanoparticle geometry, imaging systems, and experimental conditions.

Similarly, US and CEUS imaging showed significant signal enhancement in a concentration-dependent manner (Figure 5B), confirming the dual PA/CEUS imaging capacity of LQPO. Encouraged by the *in vitro* results, we next evaluated the *in vivo* distribution and dynamic oxygenation changes in tumor-bearing mice using multispectral 3D PA reconstruction imaging. To precisely quantify the hemodynamic changes, the raw PA signals were processed using the Linear Least Squares Unmixing algorithm (Vevo Lab software). This pixel-by-pixel spectral analysis enabled separation of the LQPO signal from endogenous chromophores, demonstrating the spatial distribution of LQPO (green), OxyHb (red), and DeoxyHb (blue). HbT, defined as the sum of OxyHb and DeoxyHb, was used as an indicator of local blood volume and perfusion, while sO_2_ was defined as the percentage of OxyHb relative to HbT. HbT and sO_2_ were automatically calculated using the ROI analysis tool in Vevo Lab software. As shown in Figure 5C-D, a pronounced LQPO signal was detected at the tumor site at 1 h post-injection, and this time point was therefore selected for laser irradiation. Upon NIR-II laser triggering, a rapid and synchronized increase in OxyHb, HbT, and sO_2_ was observed in the LQPO+NIR-II group, with all parameters reaching their maximum intensities at 2 h (1 h post-irradiation). This coordinated surge is attributable to ozone-mediated modulation of both tumor oxygenation and vascular perfusion. In contrast, the ozone-free LQP+NIR-II group, despite undergoing the same photothermal-triggered PFH phase transition, showed only negligible elevation in OxyHb and sO_2_ levels. This comparison indicates that photothermal activation or PFH gasification alone was insufficient to account for the observed reoxygenation. Importantly, this ozone-associated reoxygenation effect was sustained rather than transient, as both sO_2_ and HbT remained significantly elevated in the LQPO+NIR-II group throughout the 24 h observation period compared with the other groups. Detailed statistical comparisons at each time point are provided in Table S1. The strong temporal coupling between sO_2_ and HbT further suggests that the prolonged oxygenation improvement was closely associated with enhanced functional perfusion rather than simple gas release.

To provide structural evidence for this vascular response, tumor vascular morphology was quantified by CD31 immunohistochemical staining at 24 h post-treatment (Figure S4D). While control tumors displayed constricted and sparse vessels, the LQPO+NIR-II group exhibited significant vascular remodeling, with the average minimal vessel diameter and luminal area increasing from 6 μm and 50 μm^2^ to approximately 25 μm and 250 μm^2^, respectively (Figure S4E-F). Notably, no comparable vascular dilation or remodeling was observed in the LQP+NIR-II group at the same time point, supporting that ozone was the dominant driver of these sustained structural changes. This marked expansion of the vascular lumen provides a structural basis for the increased HbT and persistent reoxygenation. The biological impact of this improved perfusion was further supported by the molecular downregulation of HIF-1α (Figure S4G-H). Analysis of tumor tissues collected at the end of the 24 h window showed that HIF-1α was most significantly suppressed in the LQPO+NIR-II group, whereas the LQP+NIR-II group and the other control groups showed no appreciable decrease. Together, these findings support that the alleviation of cellular hypoxia was mainly driven by ozone-mediated modulation rather than the isolated photothermal effect.

Collectively, the LQPO platform converts the hypoxic tumor microenvironment into a reoxygenated and better-perfused state. This prolonged window of reduced hypoxia provides a biological rationale for the selected αPD-1 administration schedule.

To further confirm ozone release and its spatial distribution, CEUS imaging was performed after laser irradiation (Figure 5E). Tumors treated with LQPO+NIR-II and LQP+NIR-II displayed significantly stronger CEUS signals compared with other two groups, which was consistent with the photothermally induced liquid-gas phase transition of the PFH core into microbubbles. While CEUS provides excellent spatial guidance for gas generation, it cannot intrinsically distinguish between PFH-derived gas and ozone. Therefore, we used DHE staining as an oxidative-stress-associated fluorescent readout to evaluate ozone-associated redox activity within tumor tissues. As shown in Figure 5F, the LQPO+NIR-II group exhibited markedly stronger fluorescence than the other groups. Importantly, negligible fluorescence enhancement was observed in the LQP+NIR-II group, indicating that PFH phase transition and the associated photothermal activation alone did not induce substantial oxidative activity. Given that ozone is a highly reactive oxidant, the enhanced DHE-associated fluorescence provides indirect but biologically relevant evidence that the CEUS-visible phase transition in the LQPO system was accompanied by the release of chemically active ozone within the tumor microenvironment. Collectively, these findings demonstrate that LQPO enables reliable multimodal imaging (PAI/CEUS) to guide and monitor therapy, while simultaneously enhancing intratumoral oxygenation, thereby establishing a mechanistic link between its therapeutic activity and potential to boost immunotherapeutic efficacy.

### *In vivo* therapeutic efficacy of NIR-II laser-activated LQPO

**Figure 6:**
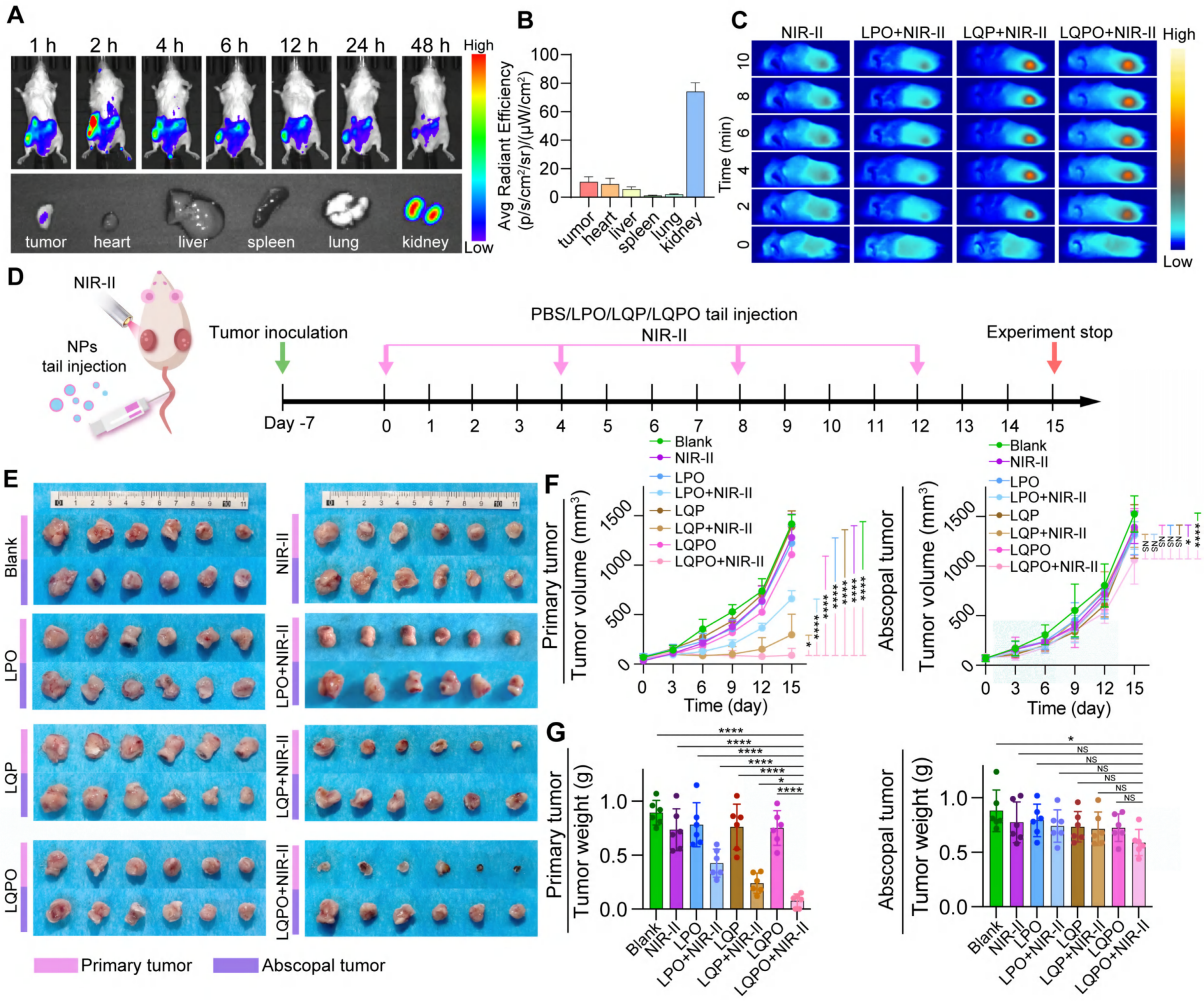
***In vivo* therapeutic efficacy of NIR-II laser-activated LQPO.** (A) Upper panel: *In vivo* fluorescence imaging showing the biodistribution of LQPO at different time points post-intravenous injection. Lower panel: *Ex vivo* fluorescence imaging of excised major organs (tumor, heart, liver, spleen, lung, and kidney) collected at the final time point, illustrating the organ-level distribution of LQPO. (B) Quantitative biodistribution analysis at 48 h. Data are presented as mean ± SD (n = 3). (C) Infrared thermal images of tumor-bearing mice during NIR-II laser irradiation (1064 nm, 0.5 W/cm^2^, 10 min). (D) Treatment schedule of NIR-II laser-activated LQPO therapy. (E) Representative *ex vivo* photographs of tumors after treatment: primary tumors (upper panel set) and abscopal tumors (lower panel set). (F) Growth curves of both primary and abscopal tumors. Data are presented as mean ± standard deviation (SD) (n = 6). (G) Final tumor weights of both primary and abscopal tumors. Data are presented as mean ± SD (n = 6). Statistical significance is indicated as *P < 0.05, **P < 0.01, ***P < 0.001, and ****P < 0.0001.

To evaluate the *in vivo* biodistribution of LQPO, fluorescence imaging was conducted in tumor-bearing mice (Figure 6A). DiR-labeled LQPO rapidly accumulated in the tumor within 2 h post-injection, reaching the highest signal intensity at this time point, which was favorable for subsequent laser-triggered therapy. At later time points, the signal gradually decreased in tumors while becoming enriched in the kidneys, suggesting renal clearance as the main metabolic pathway. *Ex vivo* fluorescence imaging at 48 h confirmed predominant accumulation in kidneys, with lower levels in other major organs (Figure 6B), further verifying renal excretion as the primary elimination route. Although the initial hydrodynamic diameter of LQPO was approximately 200 nm, this renal signal likely reflects gradual *in vivo* dissociation of non-retained nanoparticles, during which the liposomal structure was remodeled and the embedded Ti_3_C_2_ QDs were released for subsequent renal excretion. Thus, the time-dependent fluorescence redistribution from tumor to kidney may represent a dynamic targeting, dissociation, clearance process. We then monitored the local thermal effect of NIR-II laser irradiation using infrared thermography (Figure 6C). Upon 10 min of irradiation, tumors in the LQPO-treated group showed the most pronounced temperature elevation, demonstrating the efficient photothermal conversion of the QD component and its suitability for photothermal-ozone synergistic therapy.

Therapeutic efficacy was next examined *in vivo*. Mice were treated according to the indicated regimens. NIR-II laser irradiation was applied exclusively to one flank tumor, while the contralateral tumor was left untreated (Figure 6D). As shown in the excised tumor images (Figure 6E), ozone-loaded liposomes (LPO+NIR-II) displayed mild tumor suppression, while photothermal liposomes (LQP+NIR-II) achieved more evident inhibition. Remarkably, the LQPO+NIR-II group exhibited the most substantial reduction in tumor size, underscoring the synergistic antitumor effect of combined photothermal and ozone therapy. The longitudinal monitoring of tumor progression (Figure 6F) further substantiated these findings. For primary tumors, the LQPO+NIR-II group achieved the most significant suppression of growth, with tumor volumes remaining markedly lower throughout the 15-day treatment period compared with control or single-modality groups. Consistently, the lowest final tumor weights were observed in the LQPO+NIR-II group (Figure 6G). Importantly, a moderate inhibitory effect was also observed in abscopal tumors following LQPO+NIR-II treatment, as evidenced by partial inhibition of tumor growth and reduced final weights. This indicates the induction of a measurable, yet incomplete, systemic antitumor immune response, which prompted us to hypothesize that this response could be further amplified by combination with immunomodulatory agents. In summary, these results demonstrate that LQPO preferentially accumulates in tumors, can be effectively activated by NIR-II irradiation, and achieves potent local and systemic therapeutic efficacy through its synergistic photothermal and ozone-mediated mechanisms. Given its proven capacity to elicit systemic antitumor immunity, we next evaluated the synergistic potential of combining LQPO+NIR-II therapy with αPD-1 immune checkpoint blockade for superior control of both primary and abscopal tumors.

### NIR-II-activated LQPO potentiates αPD-1 therapy and elicits systemic antitumor immunity

**Figure 7:**
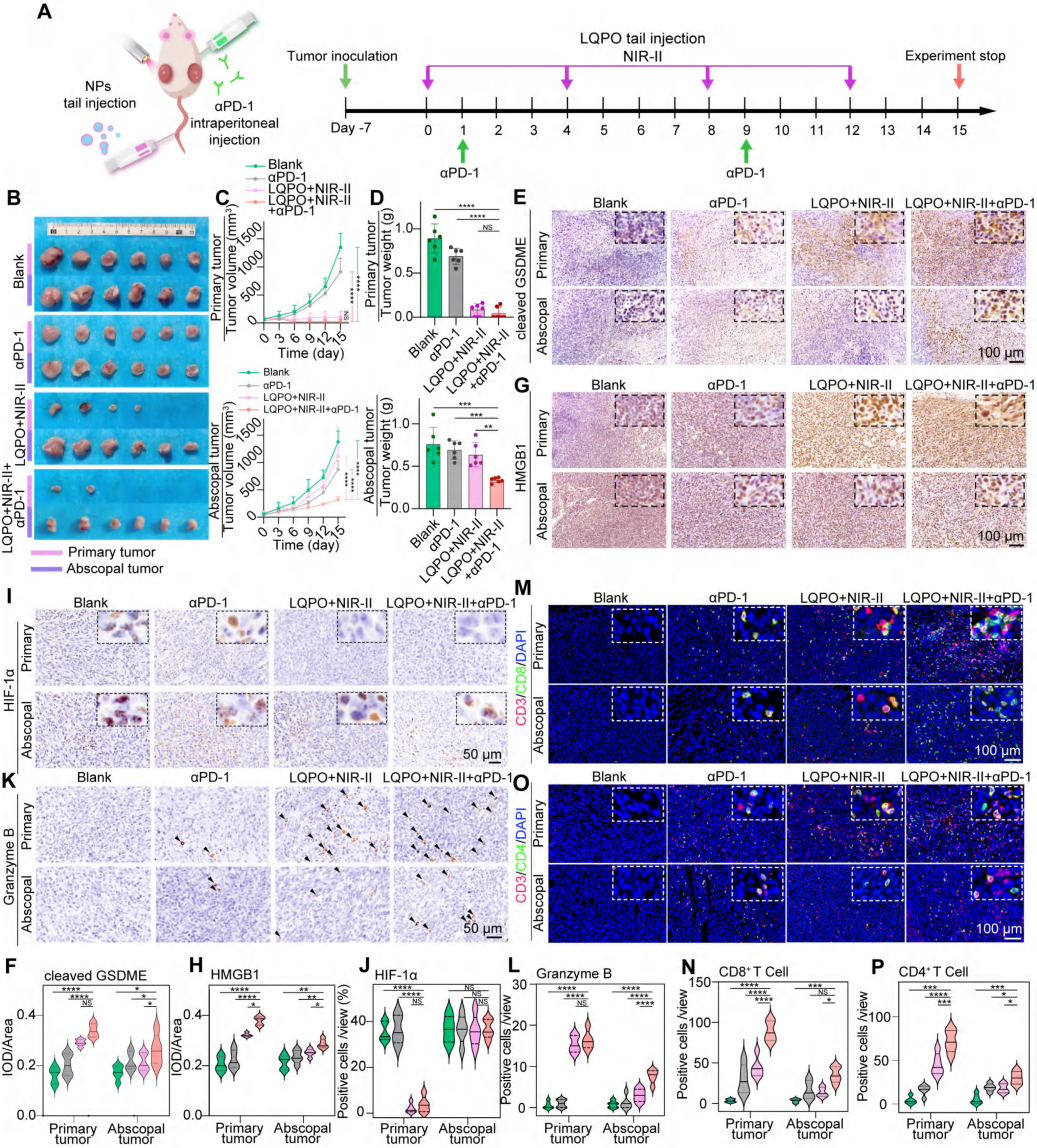
** NIR-II-activated LQPO enhances αPD-1 immunotherapy through immunogenic cell death (ICD) induction and systemic immune activation.** (A) Treatment schedule of NIR-II laser-activated LQPO therapy combined with αPD-1 therapy. (B) Representative images of bilateral 4T1 tumor-bearing mice after 15 days of treatment with phosphate-buffered saline (PBS, Blank), αPD-1, LQPO+NIR-II, and LQPO+NIR-II+αPD-1. (C) Growth curves of primary and abscopal tumors during the treatment period. Data are presented as mean ± standard deviation (SD) (n = 6). (D) Bilateral tumor weights at endpoint. Data are presented as mean ± standard deviation SD (n = 6). (E, F) Immunohistochemical (IHC) staining and quantitative analysis of cleaved gasdermin E (cleaved GSDME) in primary and abscopal tumors. Violin plots show the distribution of individual values (n = 4 per group). (G, H) IHC staining and quantitative analysis of high mobility group box 1 (HMGB1) in primary and abscopal tumors. Violin plots show the distribution of individual values (n = 4 per group). (I, J) IHC staining and quantitative analysis of hypoxia-inducible factor 1-alpha (HIF-1α) in primary and abscopal tumors. Violin plots show the distribution of individual values (n = 4 per group). (K, L) IHC staining and quantitative analysis of granzyme B in primary and abscopal tumors. Violin plots show the distribution of individual values (n = 4 per group). (M-P) Immunofluorescence staining and quantitative analysis of CD8^+^ and CD4^+^ T cells (CD8^+^/4^+^ T cells: green; nuclei: blue; CD3^+^ T cells: red) in primary and abscopal tumors. Violin plots show the distribution of individual values (n = 4 per group). Statistical significance is indicated as *P < 0.05, **P < 0.01, ***P < 0.001, and ****P < 0.0001.

To assess whether NIR-II-activated LQPO augments immune checkpoint blockade, a bilateral 4T1 model was treated with four regimens: Blank (PBS), αPD-1, LQPO+NIR-II, and LQPO+NIR-II+αPD-1 (Figure 7A). In this combination setting, αPD-1 was administered at 24 h and 9 days after the initial LQPO+NIR-II treatment. The first dose was scheduled to coincide with the early immune-priming window, during which LQPO+NIR-II had already induced marked tumor reoxygenation, improved perfusion, and ICD-associated danger signaling, thereby creating a more permissive microenvironment for T-cell activation. The second dose was given during the subsequent effector phase to sustain checkpoint blockade and reinforce systemic antitumor immunity initiated by the local *in situ* vaccination effect. After 15 days, both visual inspection and longitudinal monitoring demonstrated that the combined therapy achieved the most pronounced inhibition of both the primary and abscopal tumors (Figure 7B-D). Compared with either monotherapy, LQPO+NIR-II+αPD-1 not only produced the strongest suppression of primary tumor growth but also markedly improved control of the distant untreated tumors, indicating a more robust systemic antitumor effect. In contrast, LQPO+NIR-II alone effectively restrained the primary tumor but showed only limited activity against the abscopal tumor, whereas αPD-1 monotherapy produced only modest inhibition at both sites. Endpoint tumor weights mirrored these trends, with the smallest masses observed in the LQPO+NIR-II+αPD-1 group for both primary and abscopal tumors.

We next investigated whether the superior tumor control was accompanied by enhanced pyroptosis and ICD. Immunohistochemistry revealed markedly increased cleaved-GSDME level in the LQPO+NIR-II+αPD-1 group at both primary and abscopal tumors, indicating robust induction of pyroptotic cell death (Figure 7E-F). In parallel, strong HMGB1 nuclear depletion and extracellular release were observed (Figure 7G-H), providing direct evidence of ICD. In contrast, LQPO+NIR-II elevated cleaved-GSDME and HMGB1 release mainly in the primary tumor. Furthermore, HIF-1α staining confirmed that the ozone-mediated reoxygenation successfully alleviated cellular hypoxia in the primary tumors of both the LQPO+NIR-II and combination groups, while hypoxic regions remained prevalent in the abscopal tumors (Figure 7I-J). These findings suggest that NIR-II-triggered photothermal-ozone injury primarily induces pyroptosis, thereby initiating ICD locally, while the addition of PD-1 blockade effectively harnesses this immunogenic cascade, amplifying it and extending it to a systemic antitumor immune response.

We then examined T-cell responses within the tumor microenvironment. As a functional indicator of cytotoxic activity, Granzyme B expression was significantly upregulated in both primary and abscopal tumors of the LQPO+NIR-II+αPD-1 group, whereas the LQPO+NIR-II group showed high Granzyme B levels primarily in the primary tumor (Figure 7K-L). This systemic activation of effector function was further corroborated by T-cell infiltration patterns. In primary tumors, both LQPO+NIR-II and LQPO+NIR-II+αPD-1 markedly increased infiltration of CD8^+^ and CD4^+^ T cells, consistent with effective local activation. In abscopal tumors, however, substantial infiltration of CD8^+^ and CD4^+^ T cells was observed only in the LQPO+NIR-II+αPD-1 group (Figure 7M-P). These results indicate that local induction of immunogenic cell death synergizes with immune checkpoint blockade to elicit durable, systemic antitumor immunity.

### Biosafety Evaluation of LQPO-Based Therapy

To evaluate the biosafety of the LQPO-mediated treatment, all mice from the two rounds of *in vivo* experiments were examined for potential systemic toxicity. Systemic tolerance was first assessed through body weight monitoring across both treatment cycles. As shown in Figure S5A-B, the body weights of mice in all groups remained stable over the 15-day therapeutic period. H&E staining of major organs revealed no significant pathological abnormalities or tissue damage in any treatment group (Figure S5C). The preservation of normal hepatic lobular architecture, intact alveolar structures, and well-defined renal glomeruli indicated that neither LQPO alone nor in combination with αPD-1 therapy induced noticeable organ toxicity. To further assess systemic safety, comprehensive blood analyses were conducted. As shown in Figure S5D, key liver and kidney function indicators (ALT, AST, CREA, γ-GT, and UREA) remained within normal ranges across all groups, with no significant differences compared to the control group, indicating the absence of detectable hepatic or renal impairment. These biochemical findings were further substantiated by routine hematological parameters (Figure S5E) and a hemolysis assay (Figure S5F), which collectively confirmed the excellent blood compatibility and biosafety of the LQPO-based platform.

## Discussion

In this study, we engineer a multifunctional nanoplatform (LQPO) that initiates a precisely controlled therapeutic cascade upon a single NIR-II light stimulation. This “one-trigger, multiple-actions” design unifies PAI-guided navigation, dual-modal tumor ablation (PTT and gas therapy), and real-time CEUS imaging for therapeutic monitoring. Unlike systems where PTT and imaging function in parallel, the LQPO platform establishes a direct causal link: the NIR-II-induced thermal effect was not merely a terminal therapy but the specific trigger for the liquid-to-gas phase transition (ADV) of the PFH core. This transition simultaneously releases ozone for gas therapy and activates CEUS contrast, achieving a level of spatiotemporal control and functional synergy that ensures a coordinated anti-tumor assault. Crucially, the synergistic local therapy induces potent ICD, effectively sensitizing TNBC to systemic αPD-1 immunotherapy and inhibiting both primary and metastatic tumors.

A critical consideration for any stimulus-responsive nanoplatform is its post-activation fate and structural stability. Although NIR-II irradiation at 1 h post-injection induces the localized destruction of the liposomal carrier to release O_3_, our dynamic PA monitoring revealed a gradual rather than abrupt decline in the Ti_3_C_2_ QD signal. This suggests that the nanoplatform was likely sequestered within the intracellular compartments (e.g., lysosomes) or the dense tumor interstitium prior to activation, preventing an accelerated “washout” effect. The nearly identical pharmacokinetic profiles of the LQPO and LQP groups further confirm that the intensive gas release does not prematurely alter the retention or systemic clearance of the core nanoparticles. This localized disruption, spatially confined to the tumor, ensures therapeutic efficacy while maintaining a predictable metabolic pathway and excellent systemic biosafety, as evidenced by the stable renal clearance and absence of organ toxicity. PFH, as an inert perfluorocarbon, mainly serves as a transient phase-change and imaging medium; after vaporization, it is expected to diffuse into the circulation and be eliminated through the lungs. In contrast, O_3_ is highly reactive and was rapidly consumed within the tumor microenvironment through direct oxidation of surrounding biomolecules or spontaneous decomposition, generating molecular oxygen and transient ROS rather than persisting as a stable residue. Together, the differential post-activation fates of PFH, O_3_, and Ti_3_C_2_ QDs further support the controllability and biosafety of the LQPO platform.

Our work further expands the utility of MXene-based materials. While Ti_3_C_2_ is known for its high photothermal conversion efficiency in the NIR-II window, previous applications have largely confined its use to static PTT or PAI 30-33. By embedding Ti_3_C_2_ QDs within a thermally responsive liposomal system, we have transitioned these QDs from simple therapeutic agents to a primary engine that powers a complex, phase-change-driven delivery system 34, 35. Crucially, the applied power density (0.5 W/cm^2^ at 1064 nm) is significantly below the Maximum Permissible Exposure (MPE) threshold of 1.0 W/cm^2^ established by the ANSI Z136.1-2014 standards, ensuring clinical safety for skin exposure without inducing inflammation or thermal injury 36.

The robust and sustained reoxygenation observed in the LQPO+NIR-II group highlights a sophisticated ozone-mediated modulation of the tumor microenvironment that transcends simple gas delivery. Mechanistically, this profound elevation in sO_2_ and HbT was driven by a synergistic “chemical-biological” cascade. Chemically, the NIR-II triggered decomposition of O_3_ provided an immediate source of O_2_ within the hypoxic core. More importantly, from a biological perspective, ozone acts as a potent signaling modulator. Its reactive oxidative products can stimulate the release of endogenous vasodilators (such as nitric oxide) or directly relax tumor-associated constricted vessels 37, 38. Our results confirmed this transition, as evidenced by the significant expansion of vessel diameters. Notably, the ozone-free LQP group, despite undergoing an identical photothermal-triggered phase transition, fails to induce such reoxygenation. This suggests that while localized hyperthermia can transiently affect blood flow, it is insufficient to overcome the high interstitial fluid pressure and structural constriction of the TME without the biochemical stimulus provided by O_3_.

Beyond improving oxygenation, the observed downregulation of HIF-1α may also have broader implications for metabolic and myeloid immunosuppression within the tumor microenvironment. HIF-1α is a key regulator of hypoxia-adaptive glycolysis, including the expression of LDH-A, and sustained HIF-1α activation is closely associated with lactate accumulation and extracellular acidification 39, 40. Because lactate-rich acidic microenvironments are known to impair effector T-cell function and support immune evasion, the reoxygenation induced by LQPO+NIR-II may help relieve this metabolic barrier to antitumor immunity. In addition, hypoxia and HIF-1α signaling have been implicated in the recruitment and suppressive activity of myeloid-derived suppressor cells (MDSCs), partly through chemokine networks such as CCL2 and CXCL12 41. Therefore, although lactate levels and MDSC infiltration were not directly measured in the present study, the sustained reoxygenation and HIF-1α downregulation observed here are mechanistically consistent with a shift toward a less immunosuppressive tumor microenvironment.

While this study provides compelling proof-of-concept for the LQPO platform, we acknowledge several aspects that warrant further investigation to facilitate clinical translation. First, although the constituent components of LQPO (lipids, Ti_3_C_2_, and PFH) are generally considered to have good biocompatibility, the long-term metabolic fate and potential toxicity of the integrated nanocomposite require comprehensive evaluation. Future work should focus on detailed, long-term *in vivo* studies in animal models to rigorously assess the platform’s biodistribution, clearance pathways, and potential for chronic organ accumulation or toxicity, which are critical steps for any new nanomedicine seeking clinical approval. Second, the multi-step synthesis of LQPO, while reproducible at the laboratory scale, poses challenges for large-scale, standardized production under Good Manufacturing Practice guidelines. Batch-to-batch consistency in particle size, Ti_3_C_2_ QD loading, ozone payload, and overall stability will be essential for clinical use. Future research should therefore be directed toward optimizing and streamlining the manufacturing process, potentially through microfluidic or other automated assembly strategies to achieve precise, scalable, and reproducible nanoparticle fabrication. Third, from the perspective of clinical implementation, the main translational strengths of LQPO lie in its modular composition, multimodal imaging guidance, and on-demand activation under a single external trigger, which together may facilitate precise treatment planning and real-time therapeutic monitoring. Nevertheless, successful clinical deployment will also require standardization of laser dosimetry, optimization of treatment workflow, and compatibility with clinically accessible imaging and irradiation systems. Finally, although the NIR-II responsiveness and strong photoacoustic performance of the platform provide a promising basis for treating tumors beyond superficial lesions, the practical treatment of deep-seated tumors will still depend on anatomical accessibility and light-delivery efficiency. In this regard, future clinical translation may benefit from combining LQPO with interstitial fiber-based irradiation, endoscopic optical delivery, or other minimally invasive guidance strategies to fully exploit its potential for deep-seated malignancies.

## Conclusions

Collectively, these results demonstrate that NIR-II-activated LQPO, when combined with αPD-1 therapy, not only achieves potent suppression of primary tumors but also elicits strong abscopal responses. This synergy is mediated by ICD induction and enhanced T cell infiltration, particularly CD8^+^ T cell infiltration, offering a powerful strategy to overcome the intrinsic resistance of TNBC to immunotherapy.

## Supplementary Material

Supplementary figures.

Supplementary table 1: Statistical comparison of LQPO distribution, tumor perfusion, and oxygenation parameters in tumor-bearing mice.

## Data Availability

Data will be made available on request.

## Abbreviations

TNBC: Triple-negative breast cancer
LQPO: Liposome-Quantum dot-PFH-Ozone
PTT: Photothermal therapy
QDs: Quantum dots
PFH: Perfluorohexane
PA: Photoacoustic
CEUS: Contrast-enhanced ultrasound
ROS: Reactive oxygen species
ICD: Immunogenic cell death
GSDME: Gasdermin E
TME: Tumor microenvironment
ICIs: Immune checkpoint inhibitors
ATP: Adenosine triphosphate
HMGB1: High mobility group box 1
HF: Hydrofluoric acid
TPAOH: Tetrapropylammonium hydroxide
APTES: (3-aminopropyl) triethoxysilane
PBS: Phosphate-buffered saline
LQP: Liposome-Quantum dot-PFH
LPO: Liposome-PFH-Ozone
TEM: Transmission electron microscopy
MTT: Methyl thiazolyl tetrazolium
PI: Propidium iodide
Calcein-AM: Calcein acetoxymethyl ester
LDH: Lactate dehydrogenase
PARP: Poly (ADP-ribose) polymerase
DAPI: 4’,6-diamidino-2-phenylindole
WGA: Wheat germ agglutinin
LAMP1: Lysosome-associated membrane protein 1
CAV-1: Caveolin-1
PCC: Pearson’s correlation coefficient
MFI: Mean fluorescence intensity
CI: Combination Index
H&E: Hematoxylin and eosin
IHC: Immunohistochemistry
IF: Immunofluorescence
DHE: Dihydroethidium
OxyHb: Oxygenated hemoglobin
DeoxyHb: Deoxygenated hemoglobin
HbT: Total Hemoglobin
sO_2_: Oxygen Saturation
HIF-1α: Hypoxia-inducible factor 1-alpha
ALT: Alanine transaminase
AST: Aspartate transaminase
CREA: Creatinine
γ-GT: Gamma-glutamyl transpeptidase
UREA: Blood urea nitrogen
SPF: Specific pathogen-free
FBS: Fetal bovine serum
ANSI: American National Standards Institute
MPE: Maximum Permissible Exposure
MDSCs: Myeloid-derived suppressor cells
ADV: Acoustic droplet vaporization
LOD: Limit of detection
DCFH-DA: 2’,7’-dichlorodihydrofluorescein diacetate
