# Supplementary material for: Multimodal imaging-guided NIR-II photo-gas nanoplatform amplifies PD-1 blockade for synergistic TNBC therapy
Source: Theranostics. 2026 Jun 17;16(13):7571–93. doi: 10.7150/thno.133453 (PMC13295768; doi:10.7150/thno.133453)
Supplement: Supplementary file 1 — Supplementary figures. [file thnov16p7571s1.pdf]

Supplementary Figures

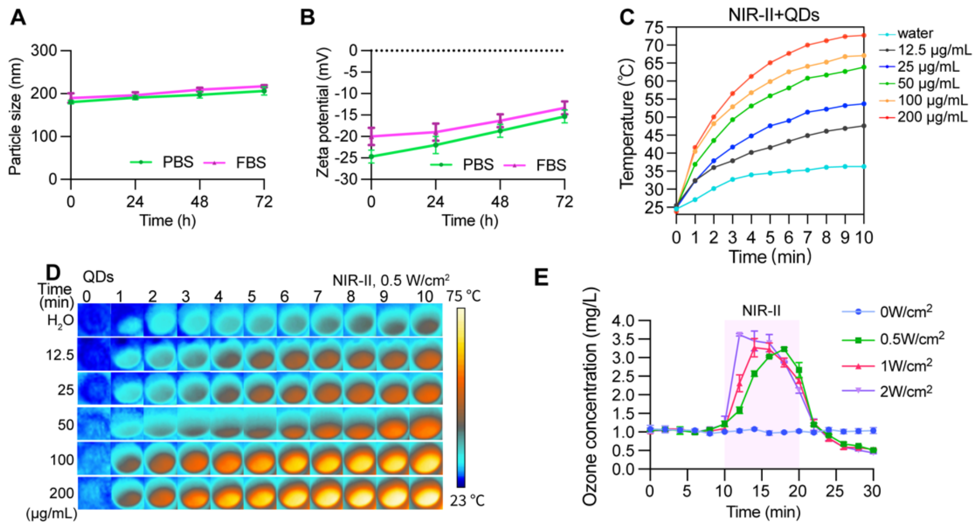

Figure S1. Colloidal stability and photothermal properties of Ti_3C_2 quantum dots (QDs) and the LQPO nanoplatform. (A, B) Changes in the hydrodynamic size and zeta potential of LQPO after incubation for 0, 24, 48, and 72 h in phosphate-buffered saline (PBS) and fetal bovine serum (FBS), respectively. Data are presented as mean ± standard deviation (SD) (n = 3). (C) Temperature elevation curves of QDs at different concentrations under NIR-II laser irradiation (0.5 W/cm^2, 10 min). (D) Infrared thermal images corresponding to (C), showing concentration-dependent photothermal heating. (E) NIR-II-triggered ozone release profiles of LQPO after different triger energy (0, 0.5, 1, and 2 W/cm^2). Data are presented as mean ± standard deviation SD (n = 3).

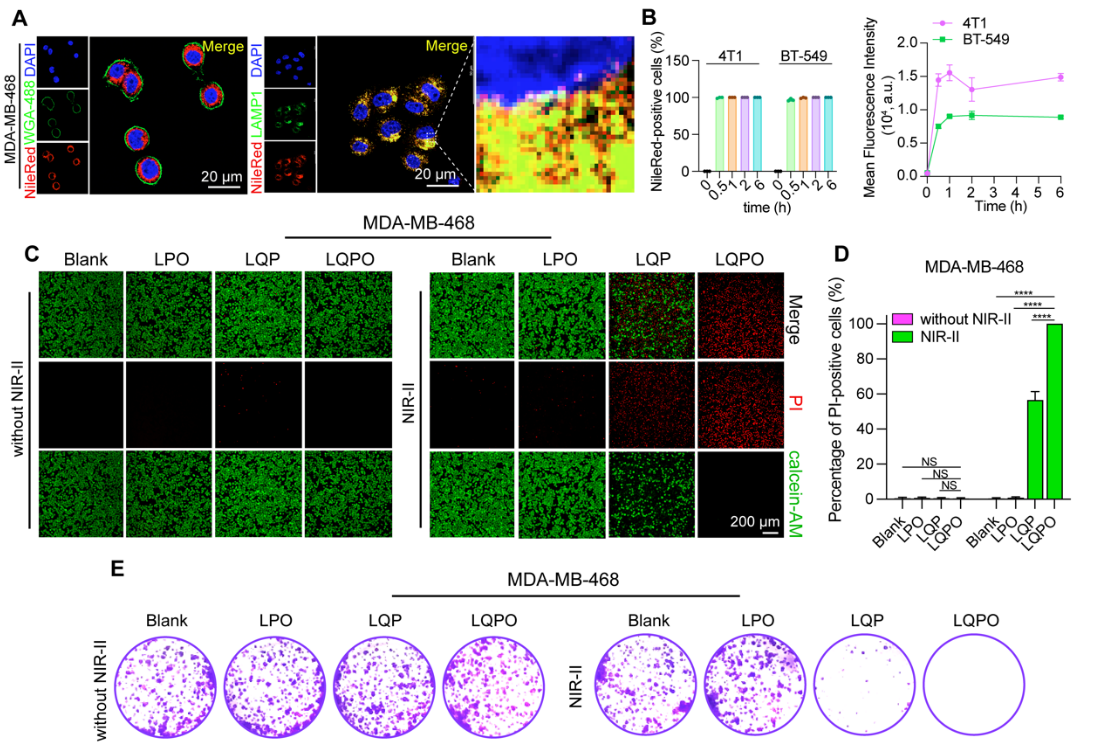

Figure S2. Cellular uptake and *in vitro* therapeutic efficacy of LQPO in MDA-MB-468 cells. (A) Confocal fluorescence images of MDA-MB-468 cells showing intracellular trafficking of Nile Red-labeled LQPO (red). Cell membranes were stained with WGA-488 (green), and colocalization with lysosomal marker LAMP1 was examined. Scale bars = 20 μm. (B) Flow cytometric quantification of Nile Red-positive cells and mean fluorescence intensity (MFI) in MDA-MB-468 cells after incubation with LQPO at the indicated time points. Data are presented as mean ± standard deviation SD (n = 3). (C, D) Live-dead staining and quantitative analysis of MDA-MB-468 cells after various treatments with or without near-infrared-II (NIR-II) irradiation. Green fluorescence indicates calcein acetoxymethyl ester (calcein-AM)-positive live cells, and red fluorescence indicates propidium iodide (PI)-positive dead cells. Scale bar = 200 μm. Data are presented as mean ± standard deviation SD (n = 4). (E) Clonogenic assays of MDA-MB-468 cells after various treatments with or without NIR-II irradiation. Statistical significance is indicated as **P* < 0.05, ***P* < 0.01, ****P* < 0.001, and *****P* < 0.0001.

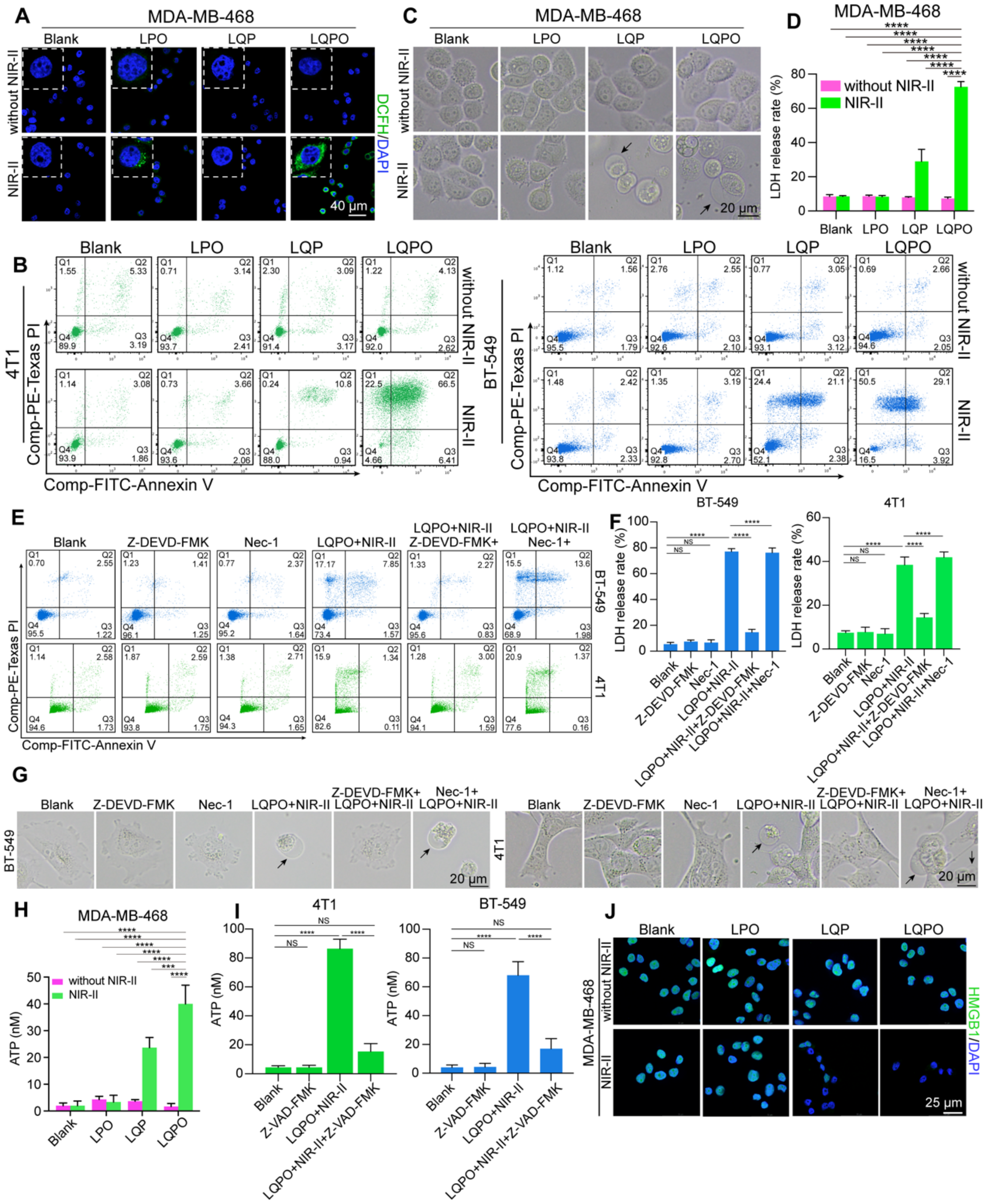

Figure S3. LQPO nanoplatform induces gasdermin E (GSDME)-dependent pyroptosis and immunogenic cell death in tumor cells. (A) Confocal fluorescence images of reactive oxygen species (ROS) generation in MDA-MB-468 cells detected by 2’,7’-dichlorodihydrofluorescein diacetate (DCFH-DA) staining after the indicated treatments with or without near-infrared-II (NIR-II) irradiation. Scale bars = 40 μm. (B) Flow cytometric analysis of Annexin V and propidium iodide (PI) staining in 4T1 and BT-549 cells after the indicated treatments, showing the distribution of viable, apoptotic, and membrane-compromised cells. (C) Representative bright-field images of MDA-

MB-468 cells after different treatment. Black arrows indicate representative cells showing swelling and ballooning morphology consistent with lytic cell death. Scale bar = 20 μm. (D) Lactate dehydrogenase (LDH) release rates in MDA-MB-468 cells after the indicated treatments, showing membrane-disruptive lytic cell death. Data are presented as mean ± standard deviation (SD) (n = 3). (E) Flow cytometric rescue analysis of Annexin V and propidium iodide (PI) staining in 4T1 and BT-549 cells after treatment with Blank, the caspase-3 inhibitor Z-DEVD-FMK, necrostatin-1 (Nec-1), LQPO+NIR-II, Z-DEVD-FMK+LQPO+NIR-II, and Nec-1+LQPO+NIR-II. (F) LDH release assay in 4T1 and BT-549 cells after treatment with Blank, the caspase-3 inhibitor Z-DEVD-FMK, necrostatin-1 (Nec-1), LQPO+NIR-II, Z-DEVD-FMK+LQPO+NIR-II, and Nec-1+LQPO+NIR-II. (G) Representative bright-field images of 4T1 and BT-549 cells after different treatment. Black arrows indicate representative cells showing swelling and ballooning morphology consistent with lytic cell death. Scale bar = 20 μm. (H) Extracellular adenosine triphosphate (ATP) release measured in cell culture supernatants after the indicated treatments. (I) Rescue analysis of extracellular ATP release in cells treated with Blank, Z-DEVD-FMK, LQPO+NIR-II, and Z-DEVD-FMK+LQPO+NIR-II. (J) Immunofluorescence staining of high mobility group box 1 (HMGB1) in MDA-MB-468 cells after the indicated treatments. Scale bar = 25 μm. For all quantitative data, values are presented as mean ± standard deviation (SD, n=3). Statistical significance is indicated as *$P < 0.05$, **$P < 0.01$, ***$P < 0.001$, and ****$P < 0.0001$.

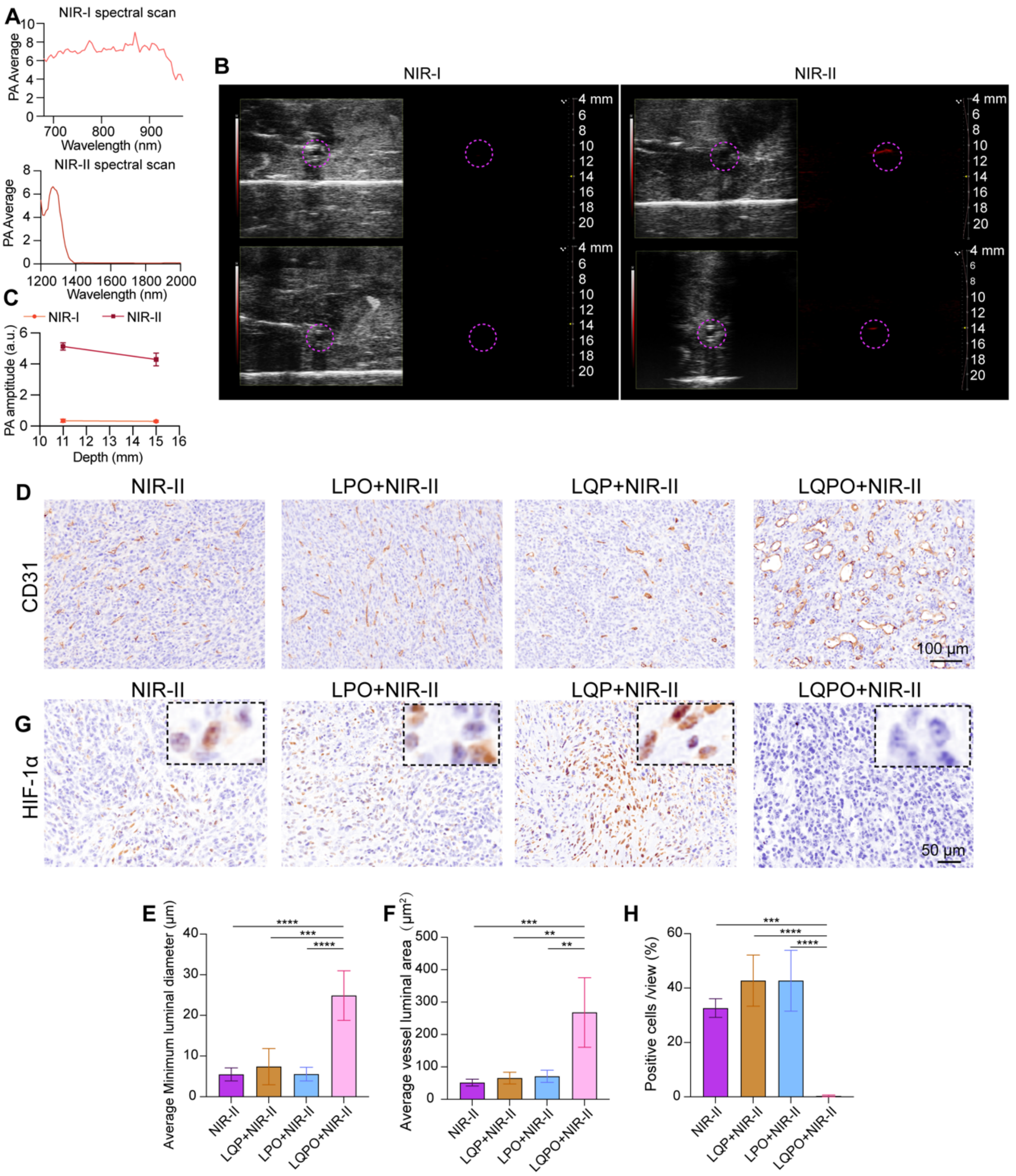

Figure S4. Near-infrared-I (NIR-I)/near-infrared-II (NIR-II) photoacoustic characteristics of LQPO and ex vivo validation of tumor hypoxia and vascular remodeling after treatment. (A) Photoacoustic spectra of the liposome-quantum dot-perfluorohexane-ozone (LQPO) nanoplatform in the NIR-I and NIR-II regions. (B) Comparison of the LQPO photoacoustic signal detected at different depth under NIR-I (750 nm) and NIR-II (1265 nm) excitation. (C) Quantitative comparison of PA signal amplitudes at different depths under NIR-I and NIR-II excitation. Data are presented as mean ± standard deviation (SD) (n = 3). (D) IHC staining of cluster of differentiation

31 (CD31) in tumor tissues collected 24 h after treatment with NIR-II alone, LPO+NIR-II, LQP+NIR-II, or LQPO+NIR-II. (E) Quantitative analysis of the average minimum vessel diameter in CD31-stained tumor sections shown in (D). (F) Quantitative analysis of the average vessel luminal area in CD31-stained tumor sections shown in (D). (G) IHC staining of HIF-1α in tumor tissues collected 24 h after the indicated treatments. (H) Quantitative analysis of HIF-1α-positive cells in tumor sections shown in (G). For all IHC quantitative data, values are presented as mean ± standard deviation (SD, n = 4). Statistical significance is indicated as $^{*}P < 0.05$, $^{**}P < 0.01$, $^{***}P < 0.001$, and $^{****}P < 0.0001$.

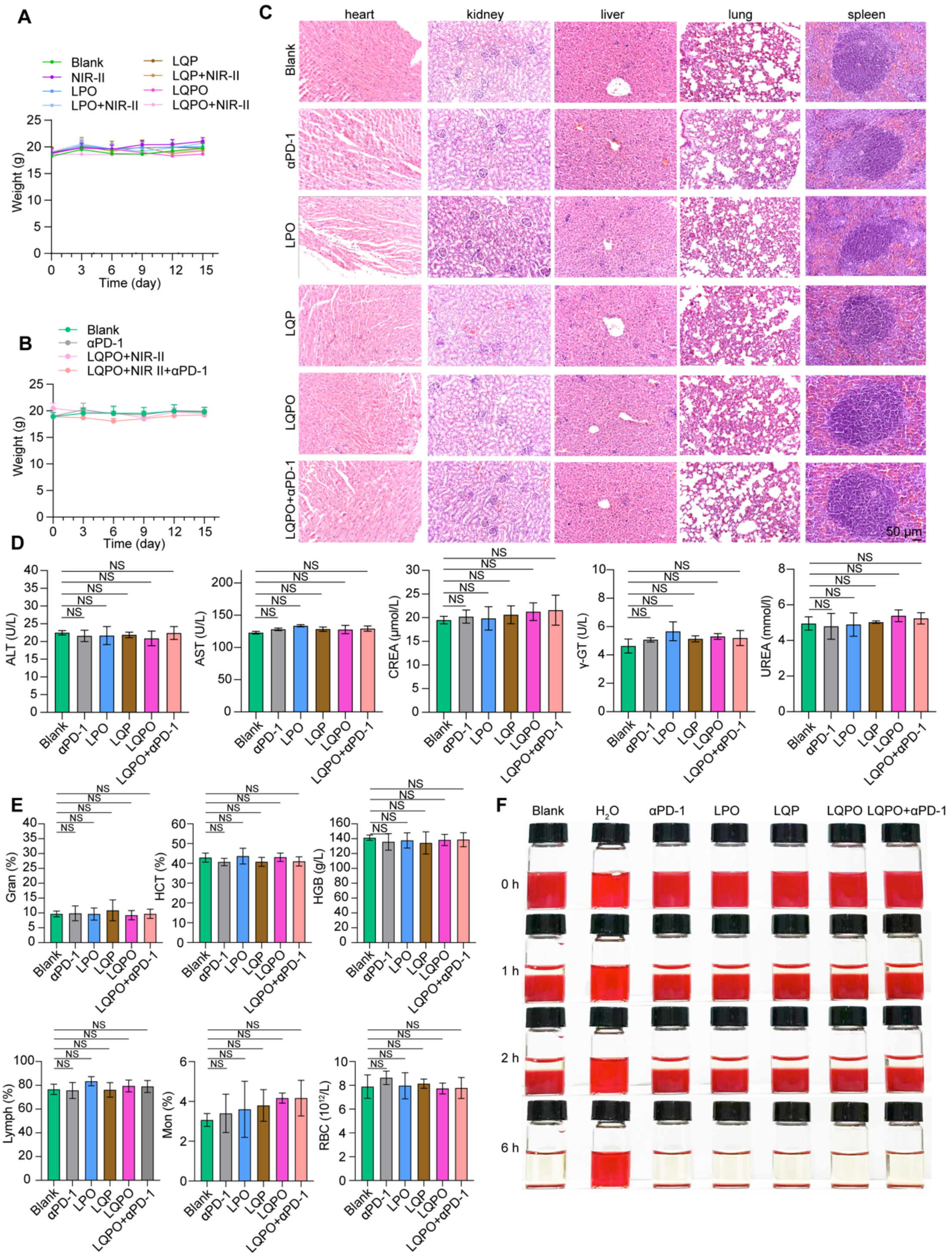

Figure S5. Biosafety evaluation of LQPO-based therapies. (A, B) Body weight monitoring during the first and second rounds of *in vivo* treatment, respectively. Data are presented as mean ± standard deviation (SD) (n = 6). (C) Representative hematoxylin and eosin (H&E)-stained images of major organs, including the heart, kidney, liver, lung, and spleen, collected from mice after treatment with the indicated formulations in both rounds of *in vivo* studies, including Blank, αPD-1, LPO, LQP,

LQPO, and LQPO+αPD-1. Scale bar = 50 μm. (D) Blood biochemical analysis of the indicated treatment groups. Data are presented as mean ± SD (n = 3). (E) Complete blood count analysis of the indicated treatment groups. Data are presented as mean ± SD (n = 3). (F) Hemolysis assay of the indicated treatment groups. Data are presented as mean ± SD (n = 3). Statistical significance is indicated as *$P < 0.05$, **$P < 0.01$, ***$P < 0.001$, and ****$P < 0.0001$.
